# Clinical value and potential mechanisms of COL8A1 upregulation in breast cancer: a comprehensive analysis

**DOI:** 10.1186/s12935-020-01465-8

**Published:** 2020-08-14

**Authors:** Wei Peng, Jian-Di Li, Jing-Jing Zeng, Xiao-Ping Zou, Deng Tang, Wei Tang, Min-Hua Rong, Ying Li, Wen-Bin Dai, Zhong-Qing Tang, Zhen-Bo Feng, Gang Chen

**Affiliations:** 1 Department of Medical Oncology, The First Affiliated Hospital of Guangxi Medical University, NO.6, Shuangyong Road, Nanning, Guangxi 530021 People’s Republic of China; 2 Department of Pathology, The First Affiliated Hospital of Guangxi Medical University, NO.6, Shuangyong Road, Nanning, Guangxi 530021 People’s Republic of China; 3 Department of Breast Surgery, Guangxi Medical University Cancer Hospital, NO.71, Hedi Road, Nanning, Guangxi 530021 People’s Republic of China; 4 Department of Research, Guangxi Medical University Cancer Hospital, NO.71, Hedi Road, Nanning, Guangxi 530021 People’s Republic of China; 5 Department of Pathology, Qinzhou First People’s Hospital, NO.8, Ming Yang Street, Qinzhou, Guangxi 535001 People’s Republic of China; 6 Department of Pathology, Liuzhou People’s Hospital, NO.8, Wenchang Road, Chengzhong District, Liuzhou, Guangxi 545006 People’s Republic of China; 7 Department of Pathology, Wuzhou Workers’ Hospital, The Seventh Affiliated Hospital of Guangxi Medical University, NO.1, Nansanxiang Gaodi Road, Wuzhou, 543000 People’s Republic of China

**Keywords:** COL8A1, Breast cancer, Immunohistochemistry staining, Mechanism

## Abstract

**Background:**

The situation faced by breast cancer patients, especially those with triple-negative breast cancer, is still grave. More effective therapeutic targets are needed to optimize the clinical management of breast cancer. Although collagen type VIII alpha 1 chain (COL8A1) has been shown to be downregulated in BRIP1-knockdown breast cancer cells, its clinical role in breast cancer remains unknown.

**Methods:**

Gene microarrays and mRNA sequencing data were downloaded and integrated into larger matrices based on various platforms. Therefore, this is a multi-centered study, which contains 5048 breast cancer patients and 1161 controls. COL8A1 mRNA expression in breast cancer was compared between molecular subtypes. In-house immunohistochemistry staining was used to evaluate the protein expression of COL8A1 in breast cancer. A diagnostic test was performed to assess its clinical value. Furthermore, based on differentially expressed genes (DEGs) and co-expressed genes (CEGs) positively related to COL8A1, functional enrichment analyses were performed to explore the biological function and potential molecular mechanisms of COL8A1 underlying breast cancer.

**Results:**

COL8A1 expression was higher in breast cancer patients than in control samples (standardized mean difference = 0.79; 95% confidence interval [CI] 0.55–1.03). Elevated expression was detected in various molecular subtypes of breast cancer. An area under a summary receiver operating characteristic curve of 0.80 (95% CI 0.76–0.83) with sensitivity of 0.77 (95% CI 0.69–0.83) and specificity of 0.70 (95% CI 0.61–0.78) showed moderate capacity of COL8A1 in distinguishing breast cancer patients from control samples. Worse overall survival was found in the higher than in the lower COL8A1 expression groups. Intersected DEGs and CEGs positively related to COL8A1 were significantly clustered in the proteoglycans in cancer and ECM-receptor interaction pathways.

**Conclusions:**

Elevated COL8A1 may promote the migration of breast cancer by mediating the ECM-receptor interaction and synergistically interplaying with DEGs and its positively related CEGs independently of molecular subtypes. Several genes clustered in the proteoglycans in cancer pathway are potential targets for developing effective agents for triple-negative breast cancer.

## Background

Breast cancer poses a grave threat to female health. According to the latest American cancer statistics, breast cancer is estimated to be the most common cancer and the second most common cause of cancer-associated deaths in women [1]. Owing to higher distal metastatic and recurrent rates, triple-negative breast cancer (TNBC) patients exhibit worse overall and disease-free survival than any other type of breast cancer [2–4]. Etiology investigations suggest that hereditary factors account for nearly one-tenth of breast cancer cases. Other risk factors, such as early or delayed menstruation, nulliparity, hormone replacement therapy, and alcohol consumption, also contribute to the prevalence of breast cancer [5]. Clinical practice guidelines recommend that females aged 40–49 or 70–74, who are at higher risk, screen for breast cancer [6]. Imaging examinations (such as bilateral breast X-ray imaging, positron emission tomography–computed tomography, and ultrasound), histological findings, and especially molecular pathology are the predominant methods of breast cancer diagnosis and assessment [7, 8]. Based on the tumor burden, optimal treatments, namely breast-conserving surgery, radiotherapy, chemotherapy, and endocrine therapy, are individually designed for breast cancer patients [6]. For TNBC patients, anthracyclines and taxanes are preferred in the initial treatment, and neoadjuvant therapy has been recognized as a standard strategy [9–12]. Unfortunately, neither endocrine therapy nor trastuzumab treatment is effective for TNBC. Targeted drugs (for example, vascular endothelial growth factor [VEGF] antibodies, epidermal growth factor receptor [EGFR] inhibitors, and mammalian target of rapamycin [mTOR] inhibitors) are gradually being employed in TNBC treatment, even though their therapeutic effects are unsatisfactory [13–16]. Therefore, the situation faced by breast cancer—especially TNBC—patients is still grave. More effective therapeutic targets are needed to optimize the clinical management of breast cancer.

Molecular events occurring in breast cancer help us better understand the onset and progression of breast cancer. It is generally agreed that chromosome 1q amplification, chromosome 16q deletion, and PIK3CA mutations are the most common pathways leading to luminal breast cancer [17, 18]. Moreover, breast cancer gene 1 (*BRCA1*) and *P53* mutations, EGFR upregulation, and cytokeratin 19 downregulation have been associated with TNBC [19, 20]. It has also been reported that circSEPT9 promotes tumor formation and TNBC progression [21]. Recently, ∆Np63 has been found to participate in breast cancer metastasis and dissemination [22]. On the other hand, several genes protect patients from breast cancer progression. For example, ZNF750, miR‑574‑5p, and circKDM4C can inhibit breast cancer progression by mediating the epigenetic regulation of pro-metastatic genes, indirectly suppressing SKIL/TAZ/CTGF and miR-548p/PBLD axis regulation [23–25]. Based on these discovered molecular mechanisms, some progress has been made in breast cancer treatment. Delivering dual microRNA using CD44-targeted mesoporous silica nanoparticles proved to be effective in TNBC treatment [26]. Although many studies provided in vitro and in vivo a detailed molecular picture of TNBC cancers [27–29], the underlying cause of breast cancer has not been fully understood. Further research is required to elucidate the breast cancer mechanisms and discover effective therapeutic targets for TNBC.

Collagen type VIII alpha 1 chain (COL8A1), also named C3orf7, is located at chromosome 3 and encodes alpha 1 chain in collagen type VIII, which is an essential component of extracellular matrix (ECM) [30]. Previous studies mainly addressed the relevance between COL8A1 and age-related macular degeneration (ADM), as well as cell proliferation [31–33]. Recently, limited studies demonstrate the de-regulation of COL8A1 in various cancers. Elevated COL8A1 expression was found in gastric cancer patients and higher COL8A1 correlated with advanced tumor stages and worse overall survival condition; and COL8A1 was selected as a candidate diagnostic biomarker in gastric cancer [34–36]. Additionally, upregulation of COL8A1 was also reported in adamantinomatous craniopharyngioma [37]. Furthermore, COL8A1 proved to participate in the progression of colon adenocarcinoma possibly by mediating focal adhesion-related pathways [38]. COL8A1 upregulation induced by TGF-β1 was found in renal cell carcinoma carcinogenesis and also correlated with poor prognosis [39]. Moreover, elevated COL8A1 in hepatocellular carcinoma promoted tumor cells proliferation, invasion, and in vivo tumorigenicity [40]. Thus far, only few studies mentioned COL8A1 in breast cancer. COL8A1 was one of the key genes restored by epigallocatechin-3-gallate in a murine breast cancer model [41]. COL8A1 proved downregulated in both BRIP1-knockdown breast cancer cells and MCF10A CDH1-/- non-cancer breast cells [42, 43]. However, the role of COL8A1 in breast cancer remains unknown.

Considering this knowledge gap, our study aimed to explore the role of COL8A1 in breast cancer. We were focused on investigating the expression of COL8A1 messenger RNA (mRNA) in various molecular subtypes of breast cancer by analyzing gene microarray and RNA sequencing data sets. The COL8A1 protein expression level was validated by immunohistochemistry staining. We also aimed to determine prognostic value of COL8A1 in breast cancer to pave the way for future clinical applications. Moreover, we explored the molecular mechanisms of COL8A1 underlying breast cancer to improve our knowledge of breast cancer carcinogenesis and progression.

## Methods

### Expression of COL8A1 mRNA in breast cancer

We integrated gene microarrays and mRNA sequencing data downloaded from Gene Expression Omnibus, The Cancer Genome Atlas (TCGA), the Genotype-Tissue Expression, the Sequence Read Archive, ArrayExpress, and Oncomine. The search formula, based on MESH terms, was as follows: ((Breast OR mammary) AND (neoplasm OR cancer OR adenoma OR carcinoma OR tumor OR BRCA OR neoplasia OR malignant OR malignancy)). Studies were screened according to the following criteria: (i) the studied species should be *Homo sapiens*; (ii) the studied specimens should be tissue dissected from patients or healthy individuals rather than cell lines. In the case of duplicated studies or samples, the most recent version was retained. The exclusion criteria were as follows: (i) expression profiles not including COL8A1; (ii) breast cancer patients receiving hormone therapy or chemotherapy; (iii) stromal rather than epithelial tumors; and (iv) metastatic rather than primary tumors. The included data sets were carefully checked and a log2 transformation was performed if any matrices had not been normalized. Additionally, the data sets were integrated into larger matrices according to various platforms, and batch effects between studies were removed using the limma-voom package in R v3.6.1. Subsequently, COL8A1 expression values were extracted and grouped according to specimen types. Standardized mean difference (SMD) were calculated to compare the expression of COL8A1 mRNA between breast cancer patients and control samples using STATA v12.0. Heterogeneity between the included studies was assessed with the *I*^2^ statistic. Statistical significance was set to an *I*^2^ value greater than 50% with a *P*-value less than 0.05. A random effects model was used in the case of significant heterogeneity. Sensitivity analysis was performed to probe the potential source of heterogeneity, and a publication bias test was used to evaluate the stability of the SMD results. Subgroup analysis was performed to compare the COL8A1 expression levels between molecular subtypes (luminal A, luminal B, human epidermal growth factor receptor 2-positive [HER-2 +], and TNBC).

### Diagnostic value of COL8A1 in breast cancer

A diagnostic test was performed to assess the clinical significance of COL8A1 in breast cancer. Based on the expression value of COL8A1, a receiver operating characteristic (ROC) curve was plotted to compute the area under the curve (AUC) using IBM SPSS Statistics v19.0. AUC values of less than 0.7, between 0.7 and 0.9, and greater than 0.9 represented weak, moderate, and strong discriminatory capability of COL8A1, respectively, between breast cancer patients and control samples. The true positives, false positives, true negatives, and false negatives rates were calculated, and the cutoff values were identified. A summary receiver operating characteristic (sROC) curve was drawn using STATA v12.0 to assess the general discriminatory capability of COL8A1 between breast cancer patients and control samples. The significance of the area under the sROC curve was consistent with that of the ROC curve. The diagnostic odds ratio (DOR), sensitivity, specificity, positive diagnostic likelihood ratio (DLR P), and negative diagnostic likelihood ratio (DLR N) were also calculated to precisely determine the accuracy and validity of COL8A1 in distinguishing breast cancer patients from control samples.

### Prognostic value of COL8A1 in breast cancer

To explore the relation between COL8A1 mRNA expression and prognosis of breast cancer patients, information on clinicopathological parameters was collected. The independent samples t-test or one-way analysis of variance was used to identify statistically significant differences in COL8A1 expression between two or more groups. A *P*-value of < 0.05 was considered statistically significant. Kaplan–Meier curves were used to compare high and low COL8A1 expression groups in terms of survival. The log-rank test was used to determine whether the prognostic difference was statistically significant.

### Investigation of COL8A1 protein expression in breast cancer by immunohistochemistry staining

A total of 115 non-specific invasive breast carcinoma and 65 normal breast tissue specimens were obtained from the First Affiliated Hospital of Guangxi Medical University, P.R.CHINA. All patients had previously signed informed consent forms, and our research was approved by the Ethics Committee of the First Affiliated Hospital of Guangxi Medical University. The breast cancer and normal breast tissue specimens were fixed with formalin. The two steps immunohistochemistry method was used to determine the protein expression of COL8A1. The primary antibody was polyclonal Antibody to COL8A1 (concentrated 1:150 dilution) purchased from Wuhan Pujian CO.LTD. Supervision TM Mouse/Rabbit-HRP Broad Spectrum Detection System (Product No. D-3004-15) was purchased from Shanghai Long Island. The experimental procedure conformed to the manufacturer’s instructions. The patients’ clinicopathological information was analyzed to determine the relationship between COL8A1 protein expression and prognosis.

### Evaluation of genetic alteration and mutation landscapes

The cBioPortal for Cancer Genomics (http://cbioportal.org) proved to be a powerful tool and facilitated our online search and cancer genomics data analysis. Using cBioPortal, we gained insight into the genetic alterations of COL8A1 in breast cancer patients and obtained information on the association between COL8A1 alterations and breast cancer patient survival. We selected the Breast Invasive Carcinoma (TCGA, Firehose Legacy) cohort, which contains 1,108 patients, and used a method of mRNA expression z-scores relative to diploid samples (RNASeqV2 RSEM). We also considered the mutation types of COL8A1 in breast cancer patients using the Catalogue of Somatic Mutations in Cancer (COSMIC), which has been recognized as the most detailed resource for somatic mutations in cancer.

### Identification of differentially expressed genes and COL8A1 co-expressed genes in breast cancer

To gain insight into the role of COL8A1 in breast cancer, we identified DEGs and COL8A1 CEGs using the limma-voom package. The DEG criteria were as follows: (i)|log2FoldChange | > 1 and (ii) adjusted *P*-value < 0.05. The CEG criteria were as follows: (i)|relation coefficient | > 0.3 and (ii) *P*-value < 0.05. Upregulated DEGs and CEGs positively related to COL8A1 were intersected. Similarly, downregulated DEGs and CEGs negatively related to COL8A1 were intersected.

### Molecular mechanisms of COL8A1 underlying breast cancer

Overlapping genes were used to perform function enrichment to shed light on the potential mechanisms of COL8A1 underlying breast cancer. The R clusterProfiler package was used to conduct Gene Ontology (GO), Kyoto Encyclopedia of Genes and Genomes (KEGG), Disease Ontology (DO), and Reactome pathway analyses. Protein-to-protein interaction (PPI) network was constructed using STRING (https://string-db.org) to investigate protein interactions. Hub genes and functional modules were identified using Cytoscape v3.6.1. Based on 1,020 breast cancer patients, the mutation landscapes of genes clustered in essential pathways were visualized using the TCGAmutations package in R v3.6.1.

## Results

### Upregulation of COL8A1 mRNA in breast cancer

**Fig. 1:**
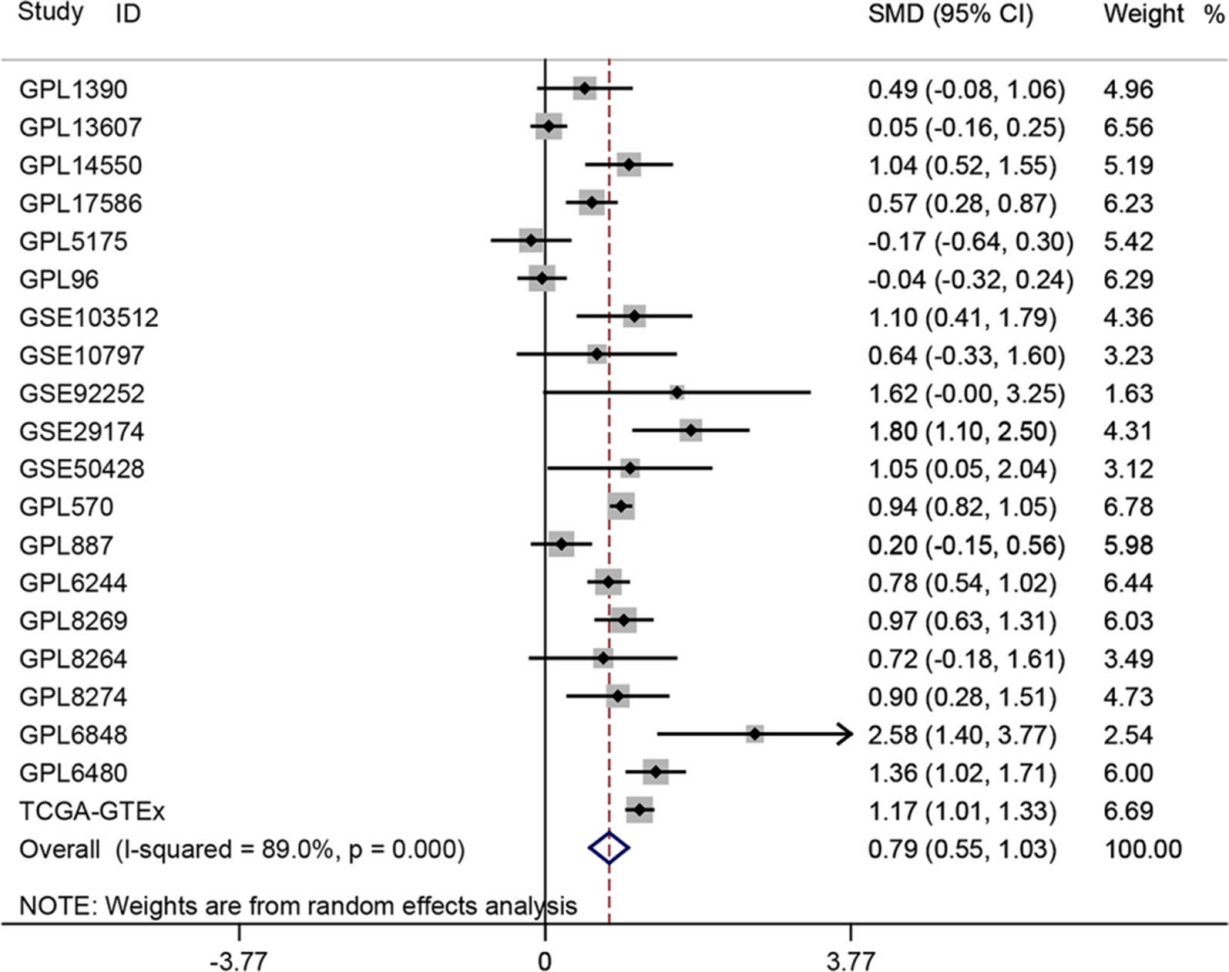
General expression status of COL8A1 in breast cancer (BRCA) compared to non-BRCA tissues. A standardized mean difference (SMD) value > 0 and 95%CI with no overlap of zero indicated COL8A1 was significantly upregulated in BRCA compared to non-BRCA tissues

**Fig. 2:**
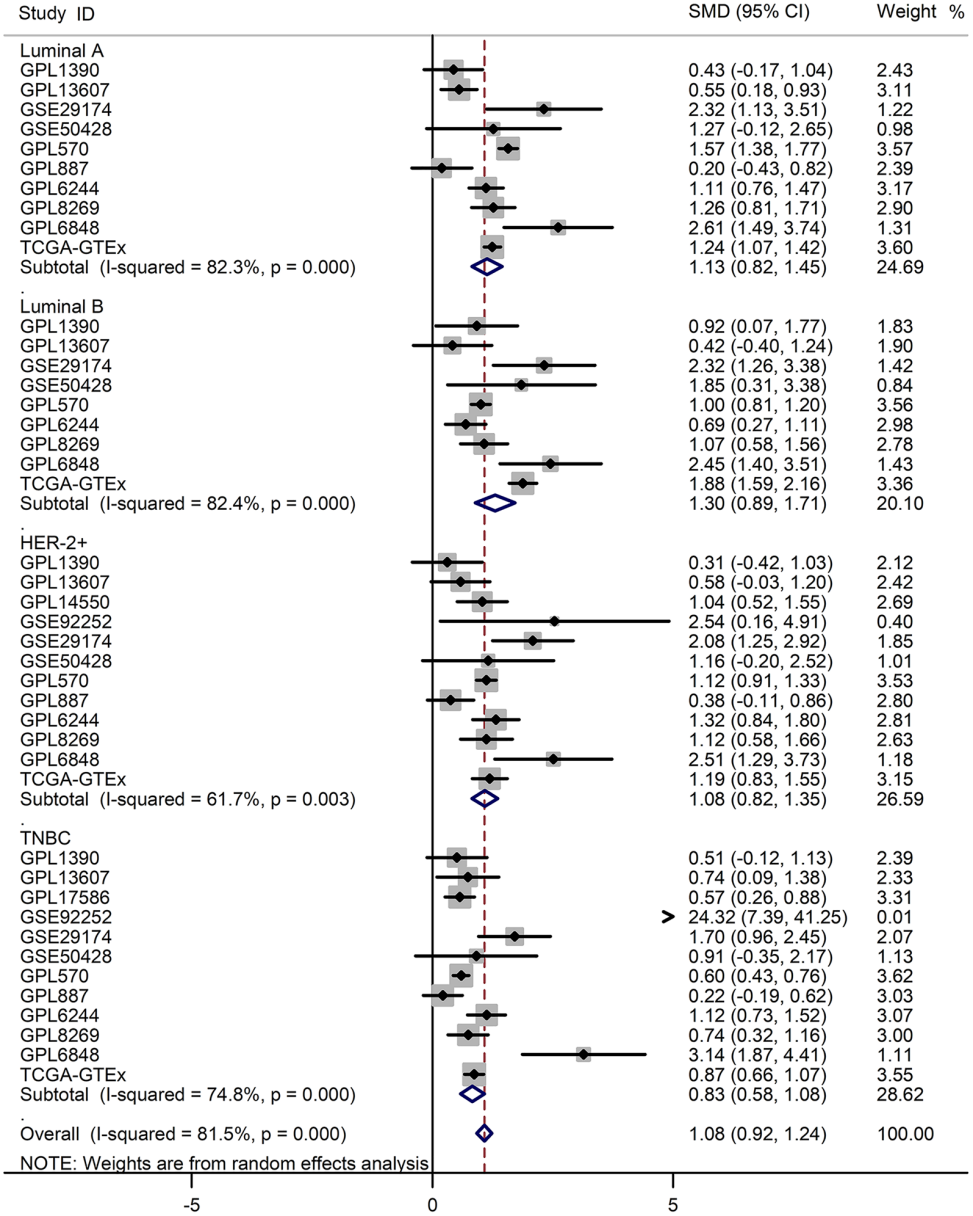
Subgroup analysis based on the subtypes of breast cancer. The result indicated that the elevated COL8A1 expression shared no significant difference among Luminal A, Luminal B, HER-2 + , and Three Negative Breast Cancer (TNBC) subgroups

Additional file 1: Figure S1 shows the flow diagram of the study inclusion process. A total of 53 studies were included and integrated into 20 larger platform matrices covering 5048 breast cancer patients and 1161 controls (Table 1). COL8A1 was generally upregulated in breast cancer compared to normal breast tissue. Thirteen of the twenty platforms showed much higher COL8A1 expression in breast cancer patients than in control samples (Additional file 2: Figure S2). Because of significant heterogeneity (*I*^2^ = 89%, *P* = 0.000), a random effects model was used. An SMD value of 0.79 (95% confidence interval [CI]: 0.55–1.03) showed that COL8A1 expression was significantly higher in breast cancer than in non-breast cancer tissue (Fig. 1). Sensitivity analysis indicated that the included studies could not explain the source of heterogeneity (Additional file 3: Figure S3a). No publication bias existed (Additional file 3: Figure S3b). Subsequently, we compared COL8A1 expression levels between different subtypes of breast cancer. COL8A1 expression was universally higher in luminal A, luminal B, HER-2 + , and TNBC patients than in control samples (Additional file 4: Figure S4, Additional file 5: Figure S5, and Additional file 6: Figure S6a). Furthermore, three platforms showed significantly higher COL8A1 expression in non-TNBC than TNBC, while only one showed lower expression in non-TNBC than TNBC (Additional file 6: Figure S6b). However, an SMD of −0.06 (95% CI −0.24–0.12) showed no difference in COL8A1 expression between TNBC and non-TNBC patients (Additional file 7: Figure S7). Subgroup analysis of four molecular subtypes of breast cancer showed no significant differences in COL8A1 expression levels between them (Fig. 2).

**Table 1:** Basic information of the enrolled data sets

Accession	BRCA			Control			t	df	*P*-*value*	TP	FP	FN	TN	GEO Series (Countries)
	N1	M1	SD1	N2	M2	SD2								
GPL1390	171	0.037	0.417	13	−0.168	0.439	−1.700	182	0.091	154	8	17	5	GSE10885; GSE10886; GSE10893; GSE1992; GSE2607; GSE6128(USA)
GPL13607	245	11.004	1.435	148	10.941	1.163	-0.478	358.939	0.633	56	15	189	133	GSE59246; GSE70951(USA)
GPL14550	48	2.483	1.162	24	1.302	1.095	−4.144	70	0	33	7	15	17	GSE33447(China); GSE83591(Canada)
GPL17586	214	2.727	0.332	57	2.540	0.300	−3.849	269	0	144	23	70	34	GSE115144(China); GSE73540(Malaysia); GSE76250(China)
GPL5175	45	4.133	0.573	28	4.224	0.463	0.707	71	0.482	20	5	25	23	GSE109169(China); GSE33692(USA)
GPL96	231	6.378	0.779	62	6.407	0.778	0.264	291	0.792	181	41	50	21	GSE15852(Malaysia); GSE5364(Singapore); GSE6883(USA)
GPL570	1611	5.576	0.813	361	4.823	0.764	−16.071	1970	0	1059	97	552	264	GSE20711(Canada); GSE45827(France); GSE65194(France); GSE29431(Spain); GSE7904(USA); GSE31448(France); GSE29044(Saudi Arabia); GSE50567(Poland); GSE61304(Singapore); GSE42568(Ireland); GSE5764(Czech Republic); GSE10780(USA); GSE10810(Spain); GSE21422(Germany); GSE22544(USA); GSE25407(USA); GSE26910(Italy); GSE54002(Singapore); GSE71053(Denmark)
GPL887	304	0.023	0.505	34	−0.081	0.585	−1.120	336	0.263	279	25	25	9	GSE10885(USA); GSE2607(USA); GSE24124 (China); GSE9309(China)
GPL6244	294	6.068	0.806	91	5.467	0.629	−7.426	189.554	0	219	32	75	59	GSE36295(Saudi Arabia); GSE86374(Mexico); GSE61724(Australia); GSE37751(USA)
GPL8269	226	2.243	0.857	41	1.434	0.676	−5.724	265	0	167	12	59	29	GSE22384-GPL8269(USA); GSE41119(USA)
GPL8264	12	1.339	1.613	9	0.303	1.185	−1.622	19	0.121	7	1	5	8	GSE22384-GPL8264(USA)
GPL8274	26	1.907	1.216	20	0.809	1.234	−3.017	44	0.004	21	5	5	15	GSE22384-GPL8274(USA)
GPL6848	19	1.400	1.125	6	−1.471	1.058	−5.521	23	0	19	1	0	5	GSE26304(Canada)
GPL6480	225	3.945	1.184	42	2.369	0.980	−8.124	265	0	195	8	30	34	GSE22820(Canada); GSE45581(USA); GSE72644(Canada)
TCGA-GTEx	1104	3.363	1.193	193	1.994	1.059	−14.936	1295	0	866	54	238	139	/
GSE103512	65	4.247	0.570	10	3.633	0.454	−3.244	73	0.002	50	1	15	9	GSE103512 (USA)
GSE10797	28	2.341	0.295	5	2.163	0.124	−1.310	31	0.2	23	1	5	4	GSE10797 (USA)
GSE92252	6	11.196	2.076	3	8.337	0.263	−3.321	5.312	0.019	6	0	0	3	GSE92252 (China)
GSE29174	148	−0.356	0.984	9	−2.102	0.701	−5.234	155	0	129	1	19	8	GSE29174 (USA)
GSE50428	26	8.519	0.809	5	7.706	0.535	−2.143	29	0.041	21	1	5	4	GSE50428 (Germany)
Total numbers	5048			1161

### Diagnostic and prognostic value of COL8A1 mRNA in breast cancer

**Fig. 3:**
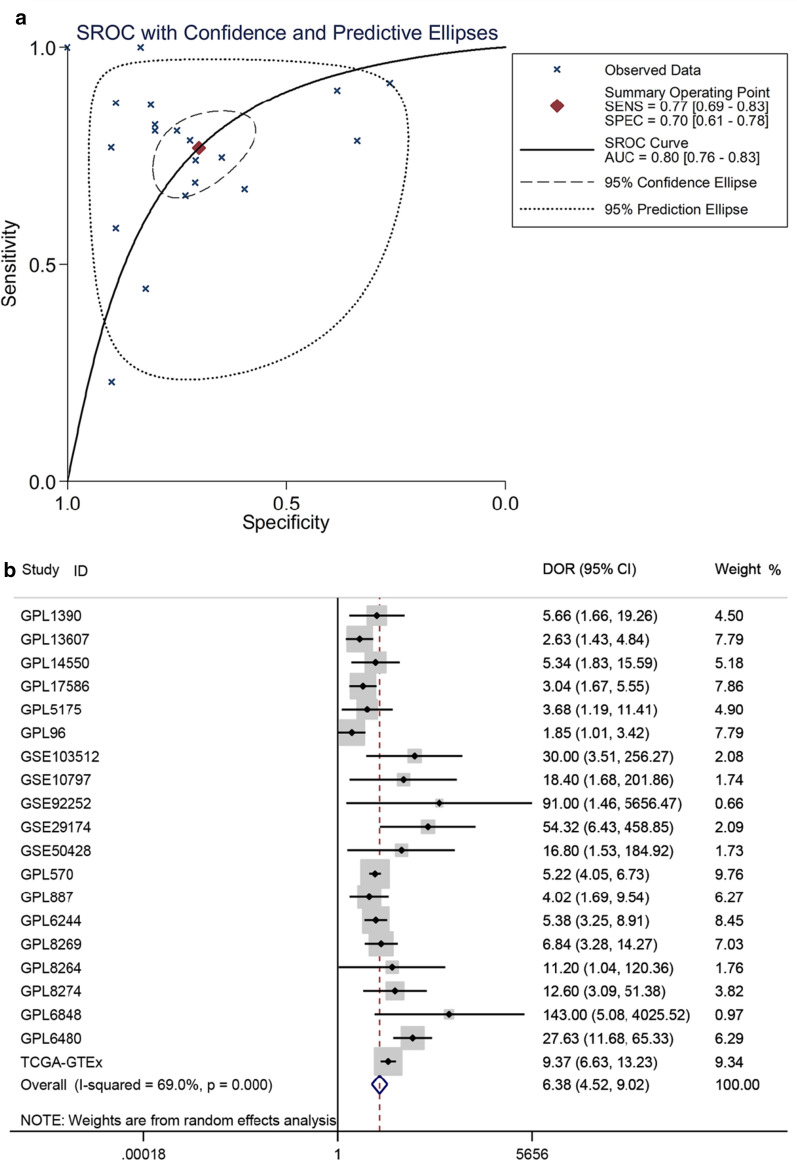
Diagnostic value of COL8A1 in breast cancer (BRCA). **a** Summary receiver operating characteristic (sROC) curve. **b** Forest plot of diagnostic odd ratio (DOR). An AUC value > 0.70 and a DOR > 1 signified COL8A1 possessed moderate capability in distinguishing BRCA from non-BRCA patients. AUC, area under the curve

**Fig. 4:**
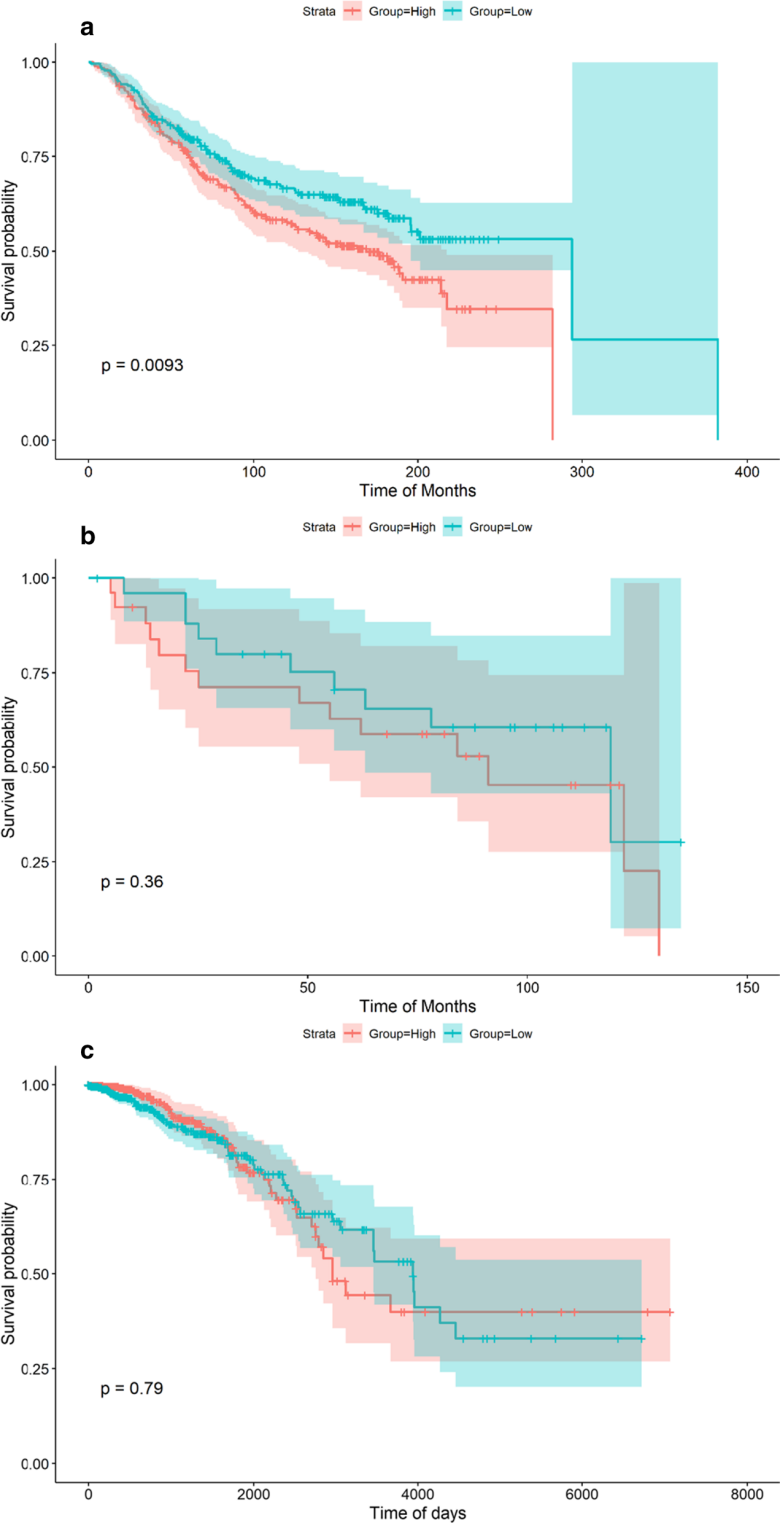
The prognostic value of COL8A1 mRNA in breast cancer tissues. **a** GSE25307; **b** GSE35629-GPL1390; **c** TCGA. In the GSE25307 cohort, high COL8A1 group possessed poor overall survival condition compared to low COL8A1 group in breast cancer patients

The clinical value of COL8A1 in breast cancer was found to be promising. Among the thirteen platforms showing high COL8A1 expression, twelve platforms indicated the ability of COL8A1 in differentiating breast cancer patients and control samples, where four platforms showed strong discriminatory capability of COL8A1 between breast cancer patients and control samples (Additional file 8: Figure S8). An area under the sROC curve of 0.80 (95% CI 0.76–0.83) with sensitivity of 0.77 (95% CI 0.69–0.83) and specificity of 0.70 (95% CI 0.61–0.78) displayed moderate capacity in distinguishing breast cancer patients from control samples (Fig. 3a). A DOR of 6.38 (95% CI 4.52–9.02) also highlighted the discriminatory ability of COL8A1 in breast cancer (Fig. 3b). The DLR P and DLR N were 2.56 (95% CI 1.96–3.33) and 0.33 (95% CI 0.25–0.44), respectively (Additional file 9: Figure S9). As shown in Additional file 10: Figure S10 and Additional file 11: Table S1, elevated COL8A1 expression correlated with race (white > black), molecular subtypes of breast cancer (luminal B > luminal A > TNBC), ER, PR, and HER-2 status. Moreover, Kaplan–Meier curves indicated worse overall survival in high compared to low COL8A1 expression groups (Fig. 4).

### Expression levels and clinical significance of COL8A1 protein in breast cancer

**Fig. 5:**
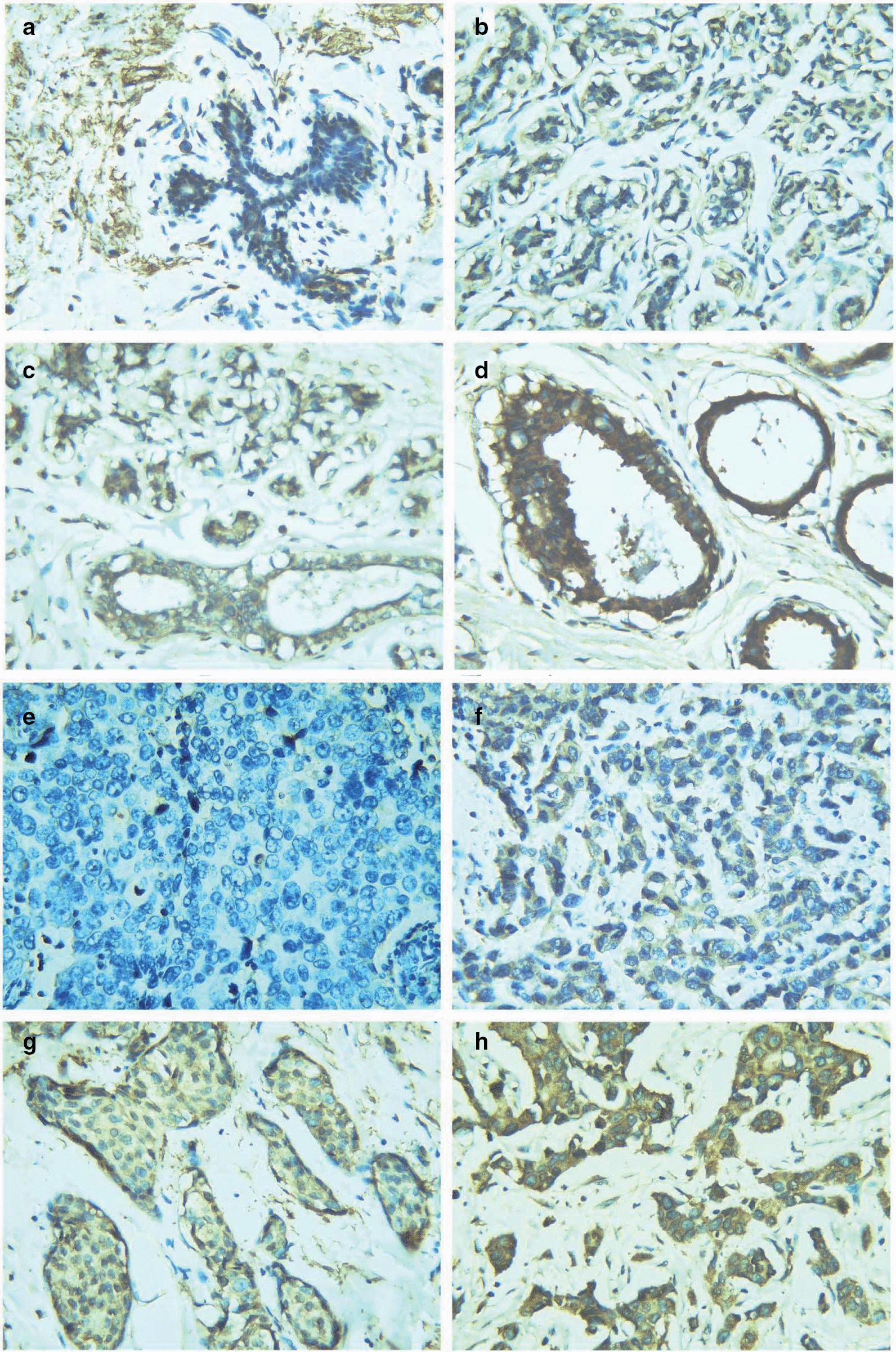
Protein expression levels of COL8A1 in breast cancer (BRCA) and normal breast tissues. **a-d** normal breast tissues; **e–h** BRCA tissues (Magnification × 200). COL8A1 was negatively stained in normal breast epithelium (**a**) and BRCA (**e**). COL8A1 was weakly stained in normal breast epithelium (**b**) and BRCA (**f**). COL8A1 was moderately stained in normal breast epithelium (**c**) and BRCA (**g**). COL8A1 was strongly stained in normal breast epithelium (**d**) and BRCA (**h**). According to staining intensity and percentage of color range, 45.2% BRCA tissues exhibited low COL8A1 expression and 54.8% BRCA tissues exhibited high COL8A1 expression. While 67.7% normal breast tissues exhibited low COL8A1 expression and 32.3% normal breast tissues exhibited high COL8A1 expression

Clinical data of breast cancer samples used to perform immunohistochemistry was summarized in Additional file 12: Table S2. Protein expression confirmed the upregulation of COL8A1 in breast cancer. The breast cancer patients whose specimens were analyzed in this study were aged between 29 and 72 (mean = 47.3), and their follow-up durations ranged from 116 to 2163 days. Immunohistochemistry staining showed varying coloration intensity of COL8A1 in breast cancer and normal breast tissue. COL8A1 was negatively, weakly, moderately, or strongly stained in normal breast epithelium (Fig. 5a–d) and breast cancer tissue (Fig. 5e–h). According to the staining intensity and color range percentages, 45.2% of breast cancer tissue specimens exhibited low and 54.8% exhibited high COL8A1 expression, whereas 67.7% of normal breast tissue specimens exhibited low and 32.3% exhibited high COL8A1 expression. A Chi square test confirmed the significantly higher expression of COL8A1 in breast cancer than normal breast tissue (*χ*^*2*^= 8.428, *P* = 0.004). Moreover, elevated COL8A1 expression correlated with estrogen-negative (ER-) breast cancer (*P* = 0.018).

### Genetic alterations and mutation kinds of COL8A1 in breast cancer

Alterations and mutations of COL8A1 in breast cancer were relatively frequent. Based on cBioPortal, COL8A1 was altered in 7% of 1108 breast cancer patients (Additional file 13: Figure S11). Amplification and high and low mRNA were the main alterations. No statistically significant difference in overall and disease-free survival was found between high and low COL8A1 expression breast cancer groups (*P* > 0.05). Furthermore, according to COSMIC, substitution missense mutations were the most frequent types (Additional file 14: Table S3).

### DEGs and COL8A1 CEGs in breast cancer

A total of 20 platform matrices were collected to determine the DEGs. The approach has been aforementioned. Initially, 3556 upregulated DEGs, 4114 downregulated DEGs, 14,467 CEGs positively related to COL8A1, and 2660 CEGs negatively related to COL8A1 were identified. Additional file 15: Figure S12 shows partial DEGs and CEGs. After being intersected, the DEGs and CEGs were divided into two gene sets: 1779 overlapping upregulated DEGs and CEGs positively related to COL8A1 (all genes appeared in no fewer than three data sets) and 322 overlapping downregulated DEGs and CEGs negatively related to COL8A1.

### Potential mechanisms of COL8A1 underlying breast cancer

**Fig. 6:**
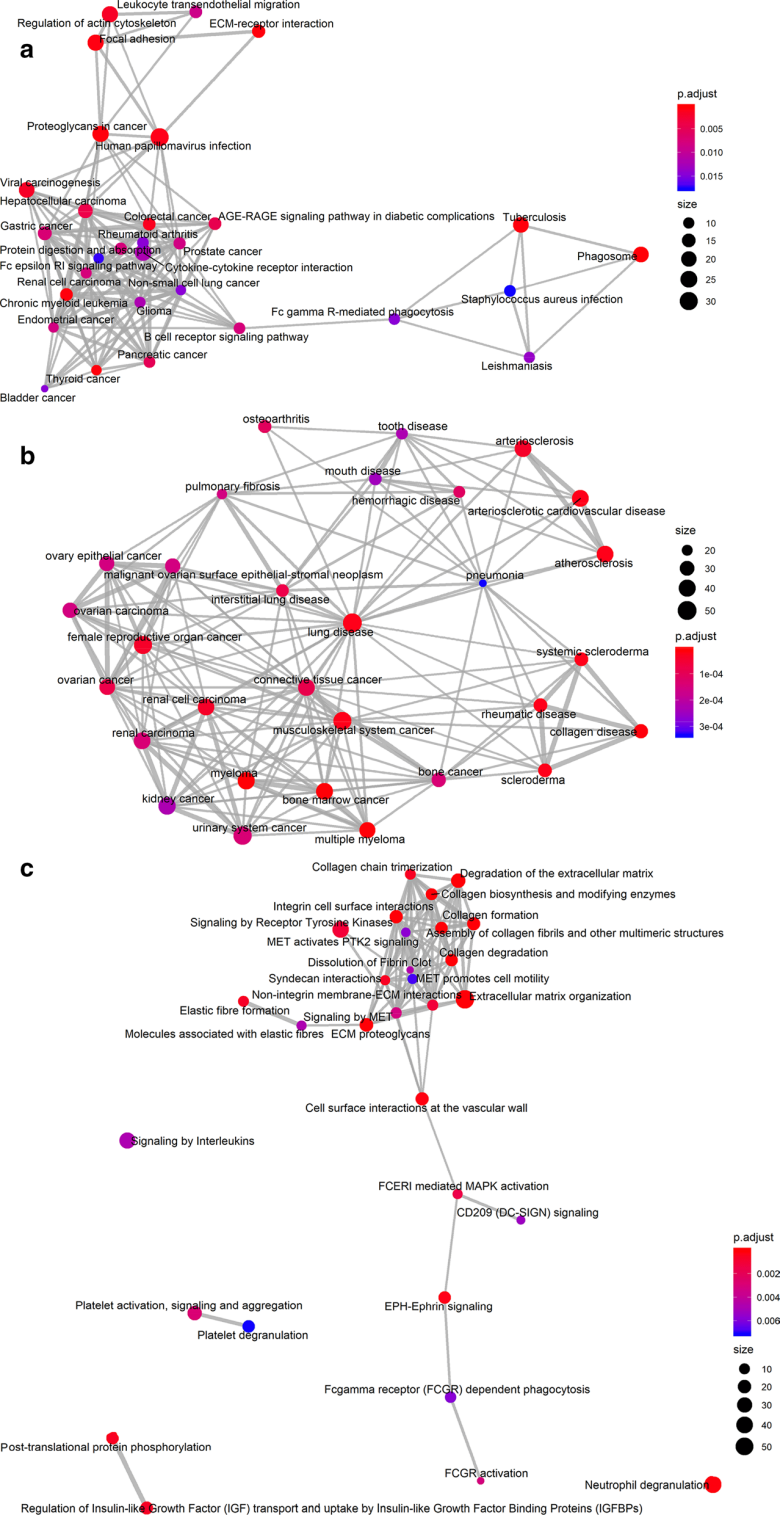
Functional enrichment based on 632 overlapping genes of upregulated DEGs and COL8A1 positively related CEGs. **a** Kyoto Encyclopedia of Genes and Genomes; **b** Disease Ontology; **c** Reactcome. DEGs, differentially expressed genes; CEGs, co-expressed genes

**Fig. 7:**
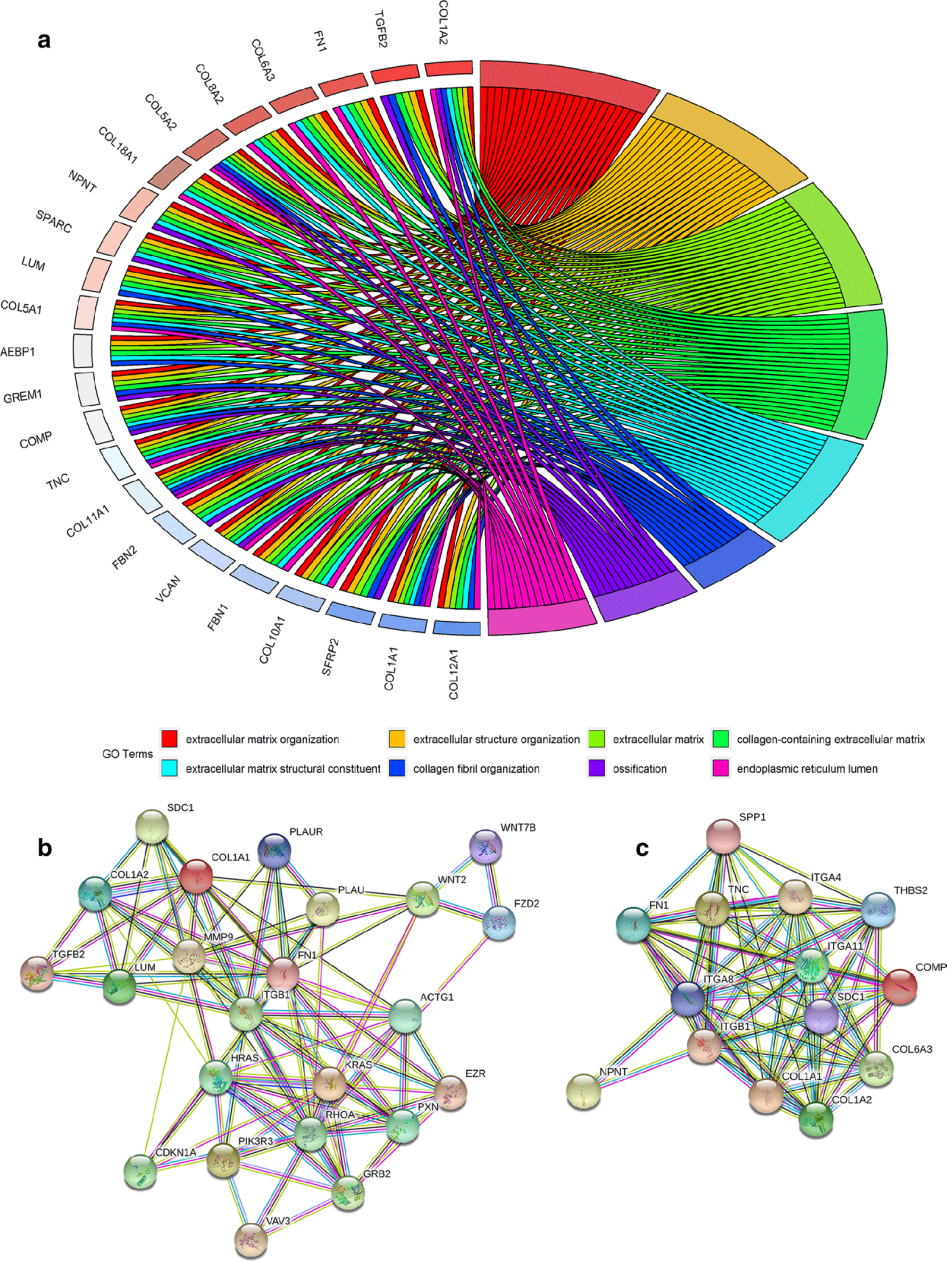
GO enrichment based on 632 overlapping genes of upregulated DEGs and COL8A1 positively related CEGs. **a** GO analysis; **b** Protein-to-protein internet based on KEGG pathway: proteoglycans in cancer (ID: hsa05205); **c** Protein-to-protein internet based on KEGG pathway: ECM-receptor interaction (ID: hsa04512). GO, Gene Ontology; DEGs, differentially expressed genes; CEGs, co-expressed genes; KEGG, Kyoto Encyclopedia of Genes and Genomes

**Fig. 8:**
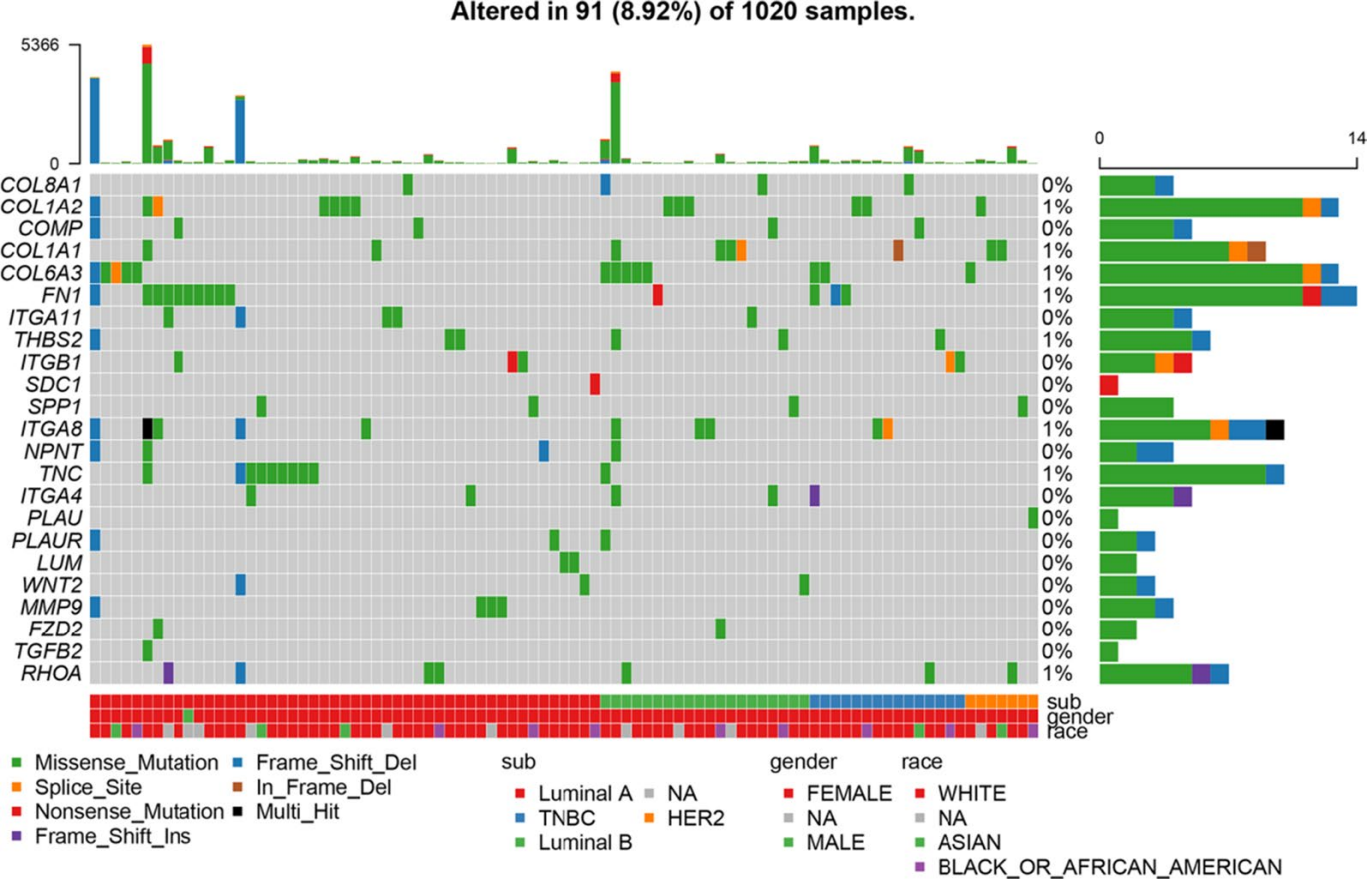
Mutation landscapes of COL8A1 and co-expressed genes clustered in two Kyoto Encyclopedia of Genes and Genomes pathways: proteoglycans in cancer and ECM-receptor interaction

The GO, KEGG, DO, and Reactome pathway analyses based on the intersected genes are shown in Additional file 16: Table S4. Regarding the 1779 overlapping upregulated DEGs and CEGs positively related to COL8A1, the following KEGG pathways were significantly aggregated (Fig. 6a): proteoglycans in cancer (Additional file 17: Figure S13), ECM-receptor interaction (Additional file 18: Figure S14), and several cancer pathways (such as thyroid cancer, colorectal cancer, and hepatocellular carcinoma). Interestingly, 14 genes (WNT2, GADD45B, FZD2, CDKN1A, KRAS, LEF1, WNT7B, BAK1, *BRCA2*, CDK4, GRB2, HRAS, PIK3R3, and POLK) were associated with the breast cancer pathway (ID: hsa05224), even though is not in the top 30 KEGG pathways. Moreover, DO analysis indicated that these genes are closely associated with myeloma and bone marrow cancer (Fig. 6b), as well as renal cell carcinoma, ovarian cancer, renal carcinoma, and other types of cancer. Furthermore, Reactome pathway analysis revealed extracellular matrix organization, ECM proteoglycans, integrin cell surface interactions, and degradation of the extracellular matrix as the top four metabolic pathways (Fig. 6c). Regarding GO enrichment, extracellular matrix organization, extracellular matrix, and extracellular matrix structural constituent were the most clustered Biological Process (BP), Cellular Component (CC), and Molecular Function (MF) terms, respectively (Fig. 7a). The proteoglycans in cancer and ECM-receptor interaction pathways were selected to construct PPI networks (Fig. 7b, c). FN1 and ITGB1 were identified as the hub genes in the two networks, respectively. The mutation landscapes of the genes in these two important pathways are shown in Fig. 8. In particular, FN1 was altered in 14 of 1020 breast cancer samples, where missense mutations accounted for 64%. The regulatory networks of COL8A1 and enriched genes in the proteoglycans in cancer and ECM-receptor interaction pathways, as well as the top two functional modules, are displayed in Additional file 19: Figure S15. On the other hand, the enrichment results regarding the 322 overlapping CEGs negatively related to COL8A1 and downregulated DEGs showed no statistical significance; these data are therefore not shown (Additional file 16: Table S5).

## Discussion

The highlight of this study is that it comprehensively explored the upregulation of COL8A1 mRNA in breast cancer from multiple aspects based on 5048 breast cancer patients and 1161 controls. Our study is multi-centered because we collected breast cancer patients from Asia, American, Europe, and Oceania, covering 16 different countries. This is the first study to investigate the protein expression of COL8A1 in breast cancer using immunohistochemistry staining. Moreover, this study is the first to assess the clinical prognostic value of COL8A1 in breast cancer. Furthermore, our study sheds light on the biological function and potential molecular mechanisms of COL8A1 underlying breast cancer.

This study demonstrates the upregulation of COL8A1 in breast cancer. We found higher COL8A1 expression in breast cancer than normal breast tissue based on 20 large platform matrices integrated from 53 data sets. Subgroup analysis based on four molecular subtypes of breast cancer showed that elevated COL8A1 expression is independent of subtypes. Further analysis also revealed universally higher COL8A1 expression levels in luminal A, luminal B, HER-2 + , and TNBC patients than in control samples. A comparison of COL8A1 expression between non-TNBC and TNBC patients showed no statistically significant difference. Immunohistochemistry staining confirmed the upregulation of COL8A1 protein in breast cancer. We thus concluded that COL8A1 expression is higher in breast cancer patients than in control samples and that upregulation is independent of molecular subtypes of breast cancer. Though the expression of COL8A1 in tissue cannot reflect the early diagnostic value in breast cancer, we assume that if COL8A1 is also differentially expressed in the bodily fluid of patients, it will be possible to serve as a potential diagnostic marker for breast cancer. Nevertheless, no previous studies have demonstrated the expression level of COL8A1 in the bodily fluid of breast cancer patients until now.

We determined the clinical value of COL8A1 in breast cancer for the first time. Our diagnostic test showed a moderate discriminatory capability of COL8A1 between breast cancer and normal breast tissue. Higher COL8A1 expression levels in breast cancer patients correlated with worse survival outcomes. Additionally, COL8A1 expression was much higher in patients of the white than of the black race. Our TCGA cohort analysis showed lower COL8A1 upregulation levels in TNBC than in the luminal A and B subtypes. Furthermore, high COL8A1 protein levels were related to ER- breast cancer. Thus, COL8A1 might serve as a prognostic marker for breast cancer.

More importantly, our study provides important clues about the role of COL8A1 in breast cancer for the first time. The intersected CEGs positively related to COL8A1 and upregulated DEGs were significantly aggregated in several cancer pathways (such as thyroid cancer, colorectal cancer, and hepatocellular carcinoma). Interestingly, we noticed that 14 genes related to COL8A1 (WNT2, GADD45B, FZD2, CDKN1A, KRAS, LEF1, WNT7B, BAK1, *BRCA2*, CDK4, GRB2, HRAS, PIK3R3, and POLK) were associated with the breast cancer pathway (ID: hsa05224), even though this pathway is not in the top 30 KEGG signaling pathways. Eight of these genes (except WNT2, GADD45B, FZD2, WNT7B, POLK, and PIK3R3) have been extensively studied in breast cancer [44–49]. Our DO analysis indicated that these genes are closely associated with myeloma, bone marrow cancer, renal cell carcinoma, ovarian cancer, and other cancer types. Therefore, searching for KEGG pathways with DO analysis, we established that COL8A1, DEGs, and positively related CEGs are associated with various cancers. Our Reactome pathway analysis showed that extracellular matrix organization, ECM proteoglycans, integrin cell surface interactions, and degradation of the extracellular matrix are the top four metabolic pathways. Extracellular matrix organization, extracellular matrix, and extracellular matrix structural constituent are the most clustered BP, CC, and MF terms, respectively. Based on these results, we selected the KEGG ECM-receptor interaction and proteoglycans in cancer pathways to construct PPI networks. ITGB1 and FN1 were identified as the hub genes, respectively. We studied the mutation landscapes of enriched genes in the proteoglycans in cancer and ECM-receptor interaction pathways. The results showed that FN1 was altered in 14 of 1020 breast cancer samples, where missense mutations accounted for 64%. The ECM is primarily composed of collagen, proteoglycans, hyaluronan, chondroitin. It is associated with tissue injury and repair, fibrosis, and tumors. Research has shown that TGF-β affects the formation of the ECM and is related to tumor cell growth and migration. For example, collagen is increased in TGF-β signaling deletion myeloid cells of mouse mammary tumor model [50]. TGF-β1 has been reported to promote the expression of COL8A1 and FN1, both of which are related to cell adhesion and the ECM [39]. Moreover, metastatic outgrowth is related to TGF-β signaling activation and FN1 upregulation induced by TGF-β [51]. In this study, we confirmed the role of FN1 as a hub gene. Furthermore, integrin plays a role in transmitting information from the ECM to cells, thus participating in cell cycle regulation and cell movement [52–54]. Our study found that COL8A1 plays a role in breast cancer. DEGs and CEGs positively related to COL8A1 are aggregated in the ECM-receptor interaction and integrin cell surface interactions. Specifically, THBS and OPN were found to be involved in cytoadhesin construction in the ECM-receptor interaction pathway. As an important component of the ECM, COL8A1 itself may interfere with signaling from the ECM to cells, affect the formation of the ECM, and promote the migration of breast cancer by synergistically interplaying with DEGs and its positively related CEGs.

The proteoglycans in cancer pathway sheds light on the special role of proteoglycans in carcinogenesis. Proteoglycans are an important component of the ECM and are associated with various cancers, such as liver, colon, and lung cancer. [55–57]. This is because when proteoglycans change dramatically, the tumor microenvironment facilitates cancer signaling and promotes tumor cell proliferation, angiogenesis, and migration [55]. Based on the proteoglycans in cancer pathway, we found that genes positively related to COL8A1 participate in the VEGF and mTOR signaling pathways, which have been identified as targets of TNBC treatments [58, 59]. For example, PI3K has been found to promote AKT and PDK-1 and eventually activate elF4B and S6 translation, thus inducing tumor cell proliferation and survival. The PI3K/AKT/mTOR pathway has been suggested as a potential target for cancer therapy [58, 59]. We found that RhoA and PI3K are associated with ECM degradation enzyme activation and cell growth, migration, and invasion in breast cancer. Interestingly, PI3K has been confirmed as a downstream target of insulin-like growth factor-1, which promotes breast cancer cell migration [60]. Therefore, COL8A1, DEGs, and positively related CEGs may be potential targets for developing more effective agents for TNBC.

Compared with other hundreds of markers already investigated in breast cancer, COL8A1 possessed several advantages. First, COL8A1 products belong to extracellular matrix protein and serve as one of the nineteen human collagens, which are important components of the breast cancer stroma. It is possible that COL8A1 participates in the communication of breast cancer cells and microenvironments [61]. Second, COL8A1 plays important role in modulating migration, proliferation, and adhesion of tumor cells [62]. Therefore, the biological function of COL8A1 makes it distinctive from other markers of breast cancer.

Certain limitations of this study should be taken into consideration. First, the discriminatory capacity of COL8A1 in breast cancer is moderate. Second, the prognostic value of COL8A1 in breast cancer needs to be confirmed by large-scale clinical practice. Third, the precise molecular mechanisms of COL8A1 in breast cancer require further examination. Fourth, the expression levels of COL8A1 in the bodily fluid of breast cancer patients need to be explored. In the future, our team may further investigate the COL8A1 expression in the bodily fluid to study its potential early diagnostic value. Finally, the roles of COL8A1, DEGs, and positively related CEGs in TNBC therapeutic strategies need to be confirmed by in vitro and in vivo experiments.

## Conclusion

This study shows that COL8A1 upregulation may promote the migration of breast cancer by mediating ECM-receptor interaction and synergistically interplaying with DEGs and its positively related CEGs independently of molecular subtypes. Several genes clustered in the proteoglycans in cancer pathway are potential targets for developing effective agents for TNBC.

## Supplementary information

**Additional file 1: Figure S1.** Flow chart of the enrolled data sets to assess the expression of COL8A1 in breast cancer.

**Additional file 2: Figure S2.** Comparison of COL8A1 expression levels between Breast cancer (BRCA) and non-BRCA tissues.

**Additional file 3: Figure S3.** Heterogeneity detection based on the enrolled data sets. **a** Sensitive analysis. The included studies were not sources of heterogeneity. **b** Funnel plot. No significant publication bias existed.

**Additional file 4: Figure S4.** Comparison of COL8A1 expression levels between: **a** Luminal A subtype breast cancer and control normal or adjacent breast cancer tissues; **b** Luminal B subtype breast cancer and control normal or adjacent breast cancer tissues.

**Additional file 5: Figure S5.** Comparison of COL8A1 expression levels between HER-2 + subtype breast cancer and control tissues.

**Additional file 6: Figure S6.** Comparison of COL8A1 expression levels between Three Negative Breast cancer (TNBC) and non-TNBC tissues.

**Additional file 7: Figure S7.** Calculation of standard mean deviation (SMD) based on COL8A1 expression in Three Negative Breast cancer (TNBC) and non-TNBC tissues.

**Additional file 8: Figure S8.** Receiver operating characteristic (ROC) curves based on COL8A1 expression value in breast cancer (BRCA) patients. An AUC value > 0.70 signified COL8A1 possessed moderate capability in distinguishing BRCA from non-BRCA patients. AUC ,area under the curve

**Additional file 9: Figure S9.** DLR positive and negative in breast cancer diagnostic trial based on COL8A1 expression level. DLR, Diagnostic likelihood ratio.

**Additional file 10: Figure S10.** Association between COL8A1 expression and clinicopathological parameters in breast cancer patients. **a** Elevated COL8A1 expression correlated with races of breast cancer patients. COL8A1 expression was higher in white compared to Black or African American. **b** Elevated COL8A1 expression correlated with subtypes of breast cancer. COL8A1 expression was lower in Three Negative Breast Cancer compared to Luminal A or Luminal B subtypes of breast cancer. **c** Elevated COL8A1 expression correlated with ER status. **d** Elevated COL8A1 expression correlated with PR status. **e** Elevated COL8A1 expression correlated with HER-2 status.

**Additional file 11: Table S1.** The relevance between COL8A1 expression and clinicopathological parameters of breast cancer patients. Independent-samples t-test or one way analysis of variance (ANOVA) was used to compare the COL8A1 expression level between two groups or more groups, respectively. ^*^A *P*-value < 0.05 indicates statistically significant.

**Additional file 12: Table S2**. Clinical data of breast cancer samples used to perform immunohistochemistry.

**Additional file 13: Figure S11.** Genetic alteration types of COL8A1 in breast cancer. **a** Amplification, mRNA high, and mRNA low were predominant alteration types of COL8A1 in breast cancer. **b**, **c**. indicated no significant difference between overall or disease-free survival conditions of COL8A1 altered and unaltered groups in breast cancer.

**Additional file 14: Table S3.** Mutation types of COL8A1 in breast cancer patients based on the Catalogue Of Somatic Mutations In Cancer (COSMIC).

**Additional file 15: Figure S12.** The identification of DEGs and COL8A1 related CEGs in breast cancer. TCGA dataset was selected to partially show A. DEGs and B. COL8A1 related CEGs. DEGs, differentially expressed genes; CEGs, co-expressed genes.

**Additional file 16: Table S4.** Functional enrichment based on 632 overlapping genes of upregulated DEGs and CEGs positively related to COL8A1. Only the top four terms or pathways were exhibited. KEGG, Kyoto Encyclopedia of Genes and Genomes; DO, Disease Ontology; GO, Gene Ontology; BP, biological process; CC, cellular component; MF, molecular function; DEGs, differentially expressed genes; CEGs, co-expressed genes. **Table S5.** Functional enrichment based on 322 overlapping genes of downregulated DEGs and CEGs negatively related to COL8A1. Only the top two terms or pathways were exhibited. KEGG, Kyoto Encyclopedia of Genes and Genomes, GO, Gene Ontology; BP, biological process; CC, cellular component; MF, molecular function.

**Additional file 17: Figure S13.** Intersected DEGs and CEGs positively related to COL8A1 clustered in the proteoglycans in cancer pathways Kyoto Encyclopedia of Genes and Genomes (KEGG) pathway: Proteoglycans in cancer (ID: hsa05205).

**Additional file 18: Figure S14.** Intersected DEGs and CEGs positively related to COL8A1 clustered in the ECM-receptor interaction pathways Kyoto Encyclopedia of Genes and Genomes (KEGG) pathway: ECM-receptor interaction (ID: hsa04512).

**Additional file 19: Figure S15**. Co-expressed network of COL8A1 in breast cancer. Genes clustered in the top two KEGG pathways or the top two functional modules were displayed. KEGG, Kyoto Encyclopedia of Genes and Genomes.

## Data Availability

The data sets used and/or analyzed during the current study are available from the corresponding author on reasonable request.

## Abbreviations

COL8A1: Collagen type VIII alpha 1 chain
DEGs: Differentially expressed genes
CEGs: Co-expressed genes
TNBC: Triple-negative breast cancer
VEGF: Vascular endothelial growth factor
EGFR: Epidermal growth factor receptor
mTOR: Mammalian target of rapamycin
*BRCA1*: Breast cancer gene 1
ECM: Extracellular matrix
ADM: Age-related macular degeneration
mRNA: Messenger RNA
SMD: Standard mean deviations
HER-2+: Human epidermal growth factor receptor 2-positive
ROC: Receiver operating characteristic
AUC: Area under the curve
sROC: Summary receiver operating characteristic
DOR: Diagnostic odds ratio
DLR P: Positive diagnostic likelihood ratio
DLR N: Negative diagnostic likelihood ratio
GO: Gene Ontology
KEGG: Kyoto Encyclopedia of Genes and Genomes
DO: Disease Ontology
PPI: Protein-to-protein interaction
CI: Confidence interval
BP: Biological process
CC: Cellular component
MF: Molecular function
