# Supplementary figures and images for: Clinical value and potential mechanisms of COL8A1 upregulation in breast cancer: a comprehensive analysis
Source: Cancer Cell Int. 2020 Aug 14;20:392. doi: 10.1186/s12935-020-01465-8 (PMC7427770; doi:10.1186/s12935-020-01465-8)

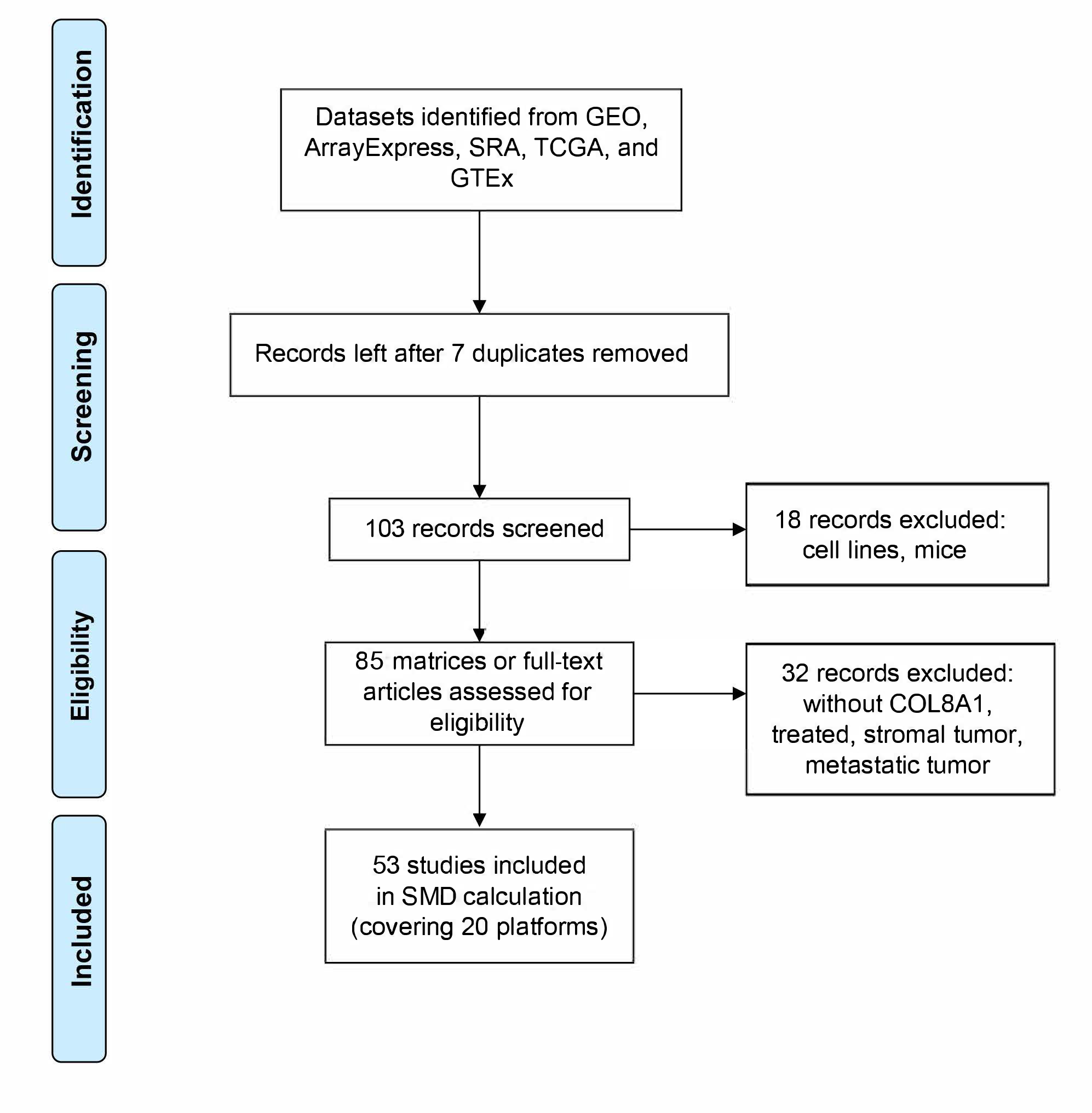

Supplement: Supplementary file 1 — Additional file 1: Figure S1. Flow chart of the enrolled data sets to assess the expression of COL8A1 in breast cancer. [file 12935_2020_1465_MOESM1_ESM.tif]

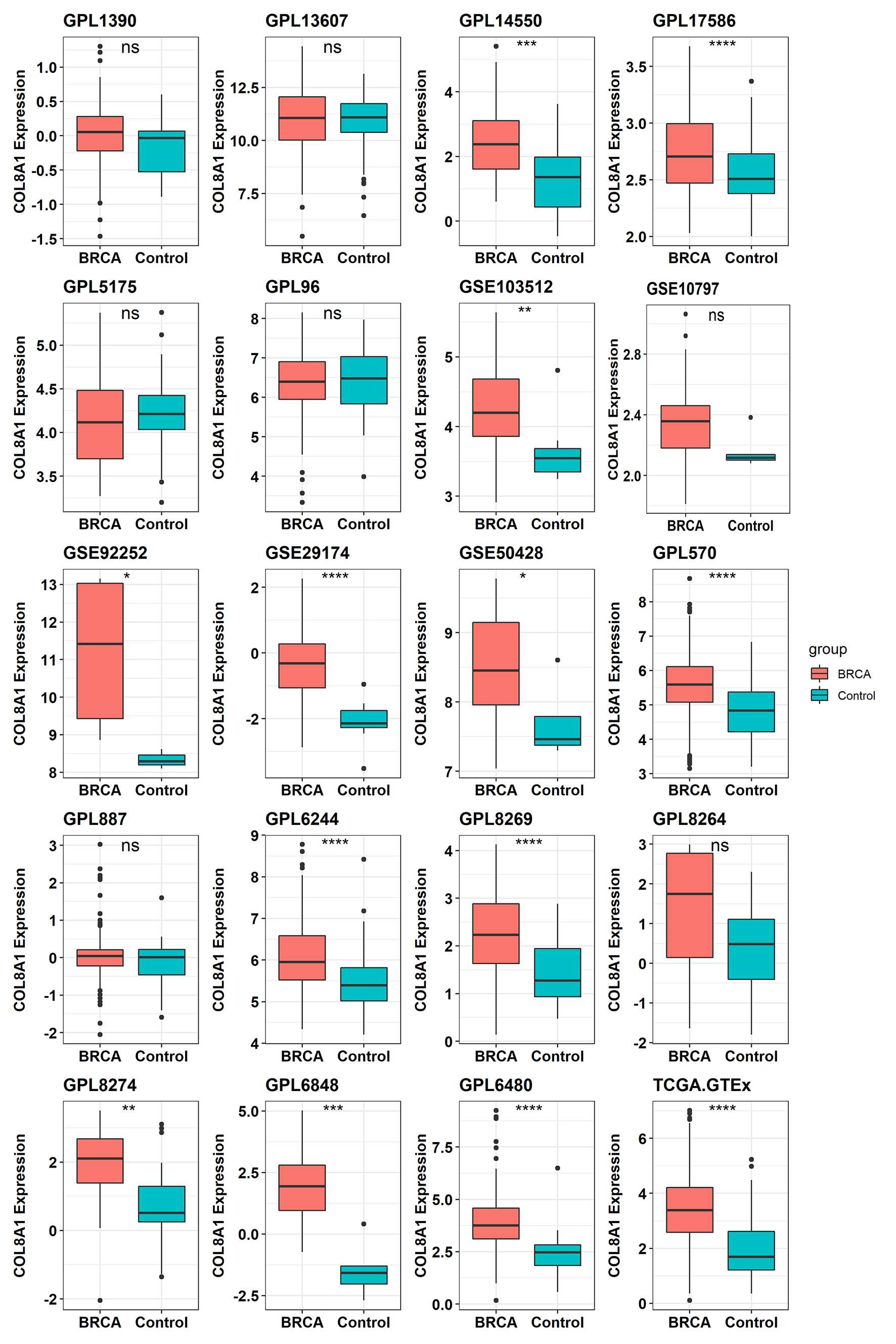

Supplement: Supplementary file 2 — Additional file 2: Figure S2. Comparison of COL8A1 expression levels between Breast cancer (BRCA) and non-BRCA tissues. [file 12935_2020_1465_MOESM2_ESM.tif]

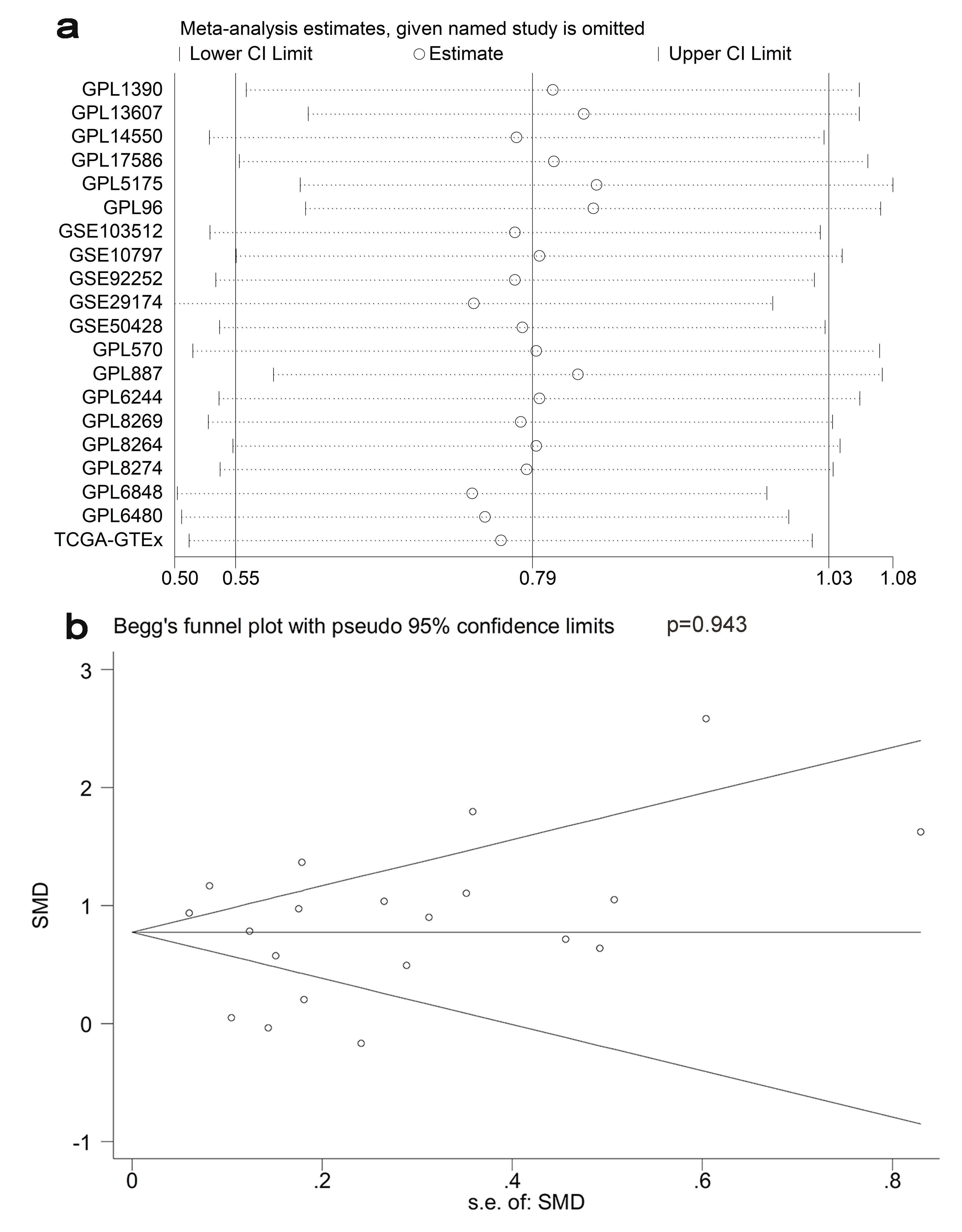

Supplement: Supplementary file 3 — Additional file 3: Figure S3. Heterogeneity detection based on the enrolled data sets. a Sensitive analysis. The included studies were not sources of heterogeneity. b Funnel plot. No significant publication bias existed. [file 12935_2020_1465_MOESM3_ESM.tif]

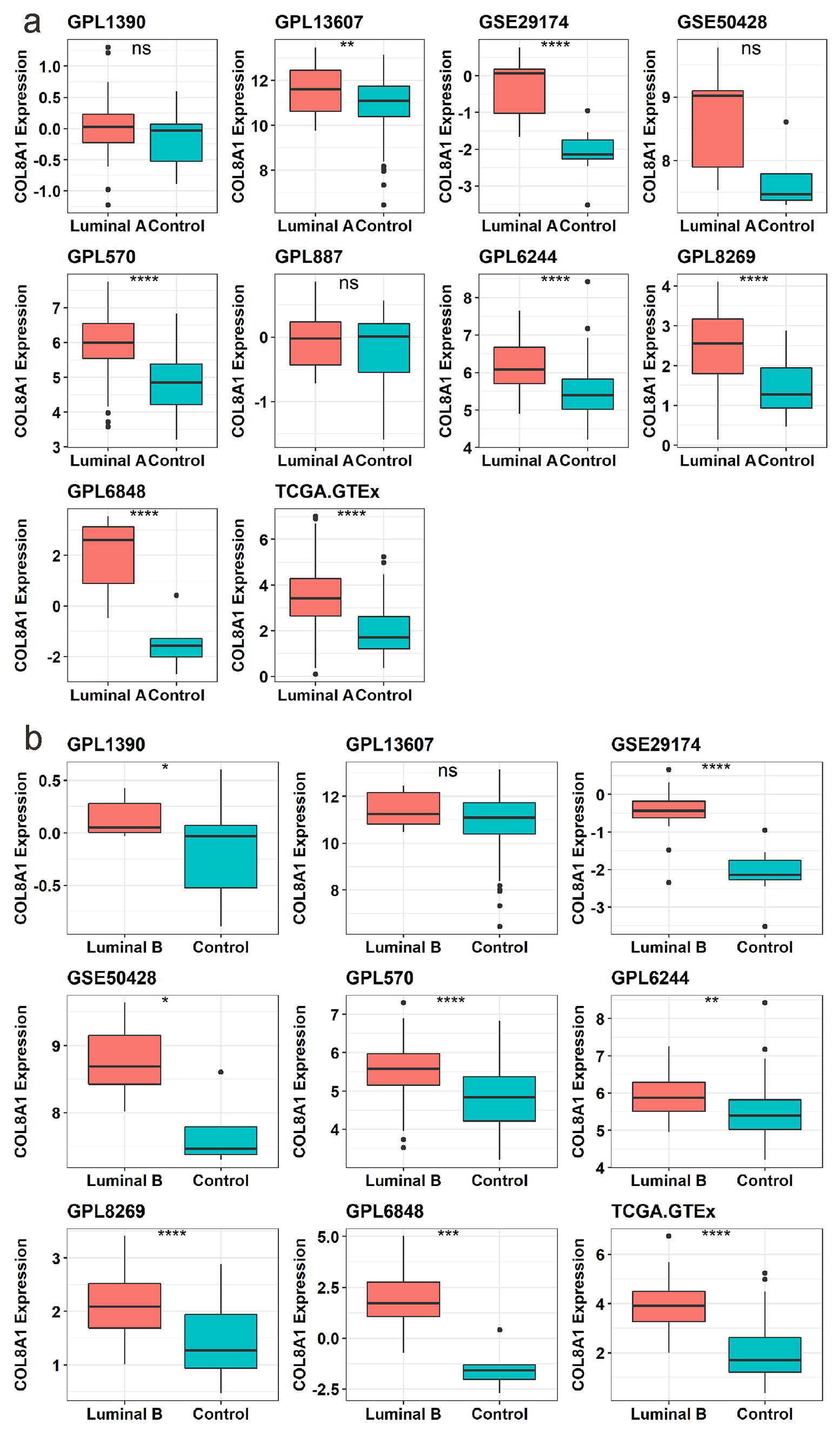

Supplement: Supplementary file 4 — Additional file 4: Figure S4. Comparison of COL8A1 expression levels between: a Luminal A subtype breast cancer and control normal or adjacent breast cancer tissues; b Luminal B subtype breast cancer and control normal or adjacent breast cancer tissues. [file 12935_2020_1465_MOESM4_ESM.tif]

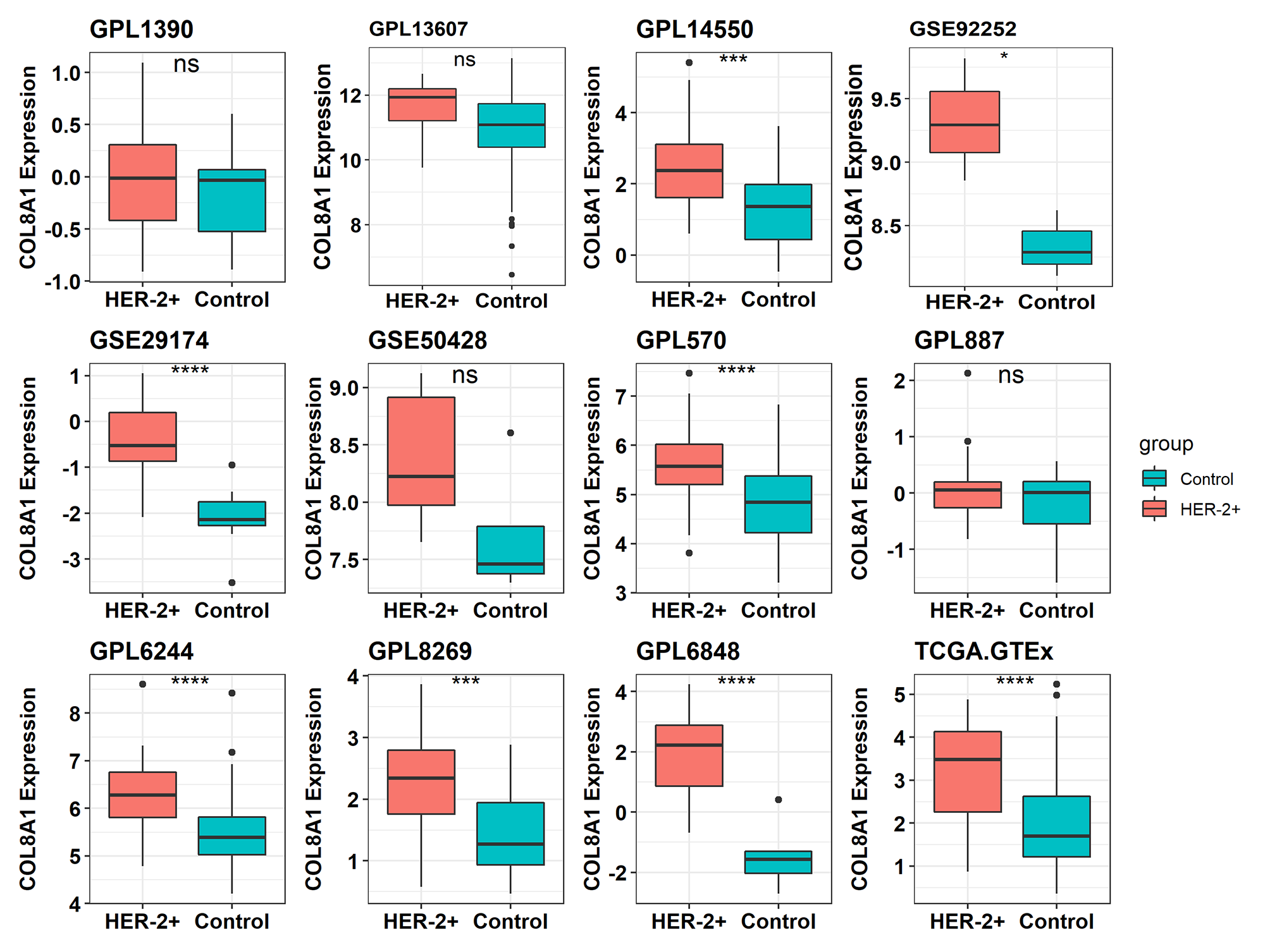

Supplement: Supplementary file 5 — Additional file 5: Figure S5. Comparison of COL8A1 expression levels between HER-2 + subtype breast cancer and control tissues. [file 12935_2020_1465_MOESM5_ESM.tif]

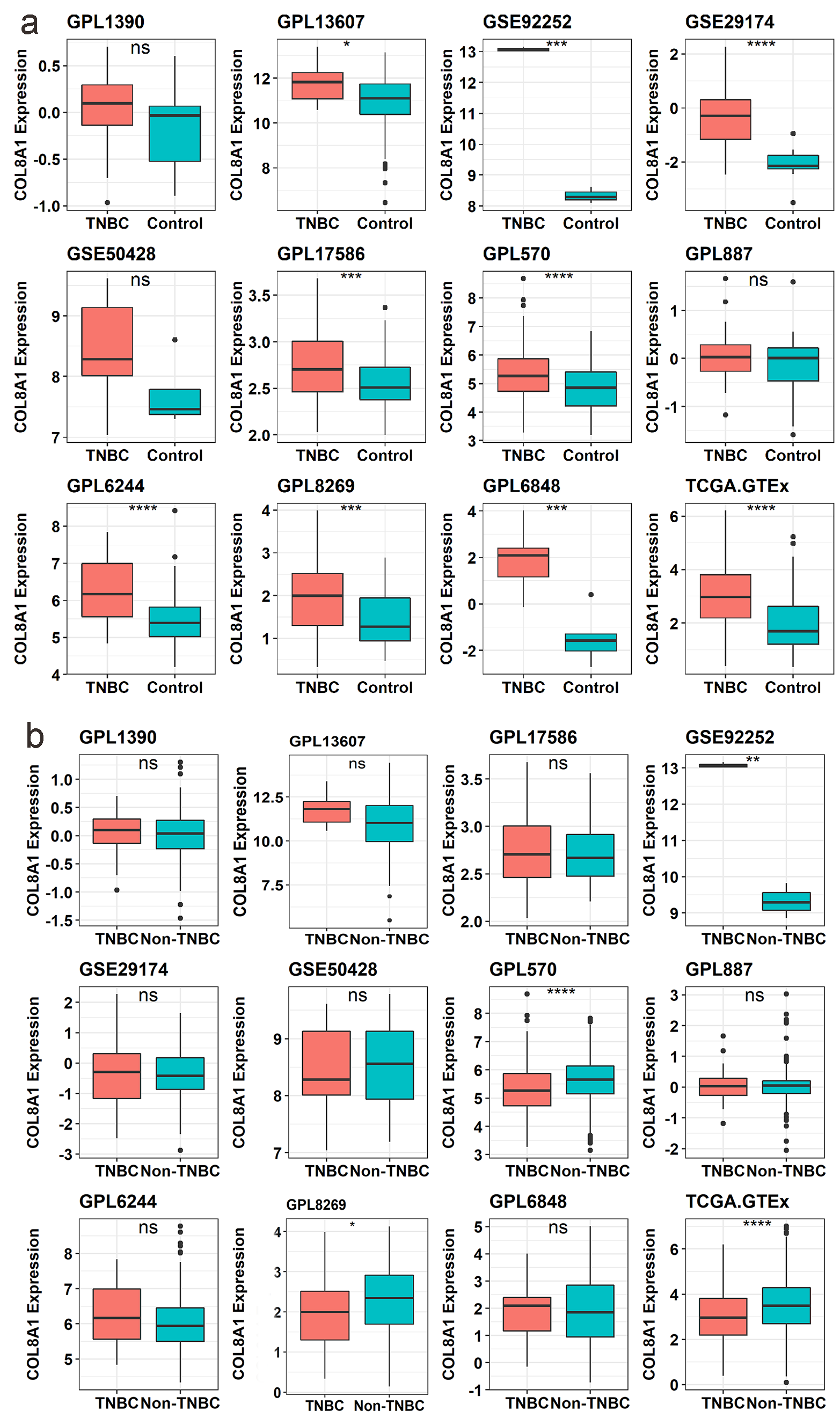

Supplement: Supplementary file 6 — Additional file 6: Figure S6. Comparison of COL8A1 expression levels between Three Negative Breast cancer (TNBC) and non-TNBC tissues. [file 12935_2020_1465_MOESM6_ESM.tif]

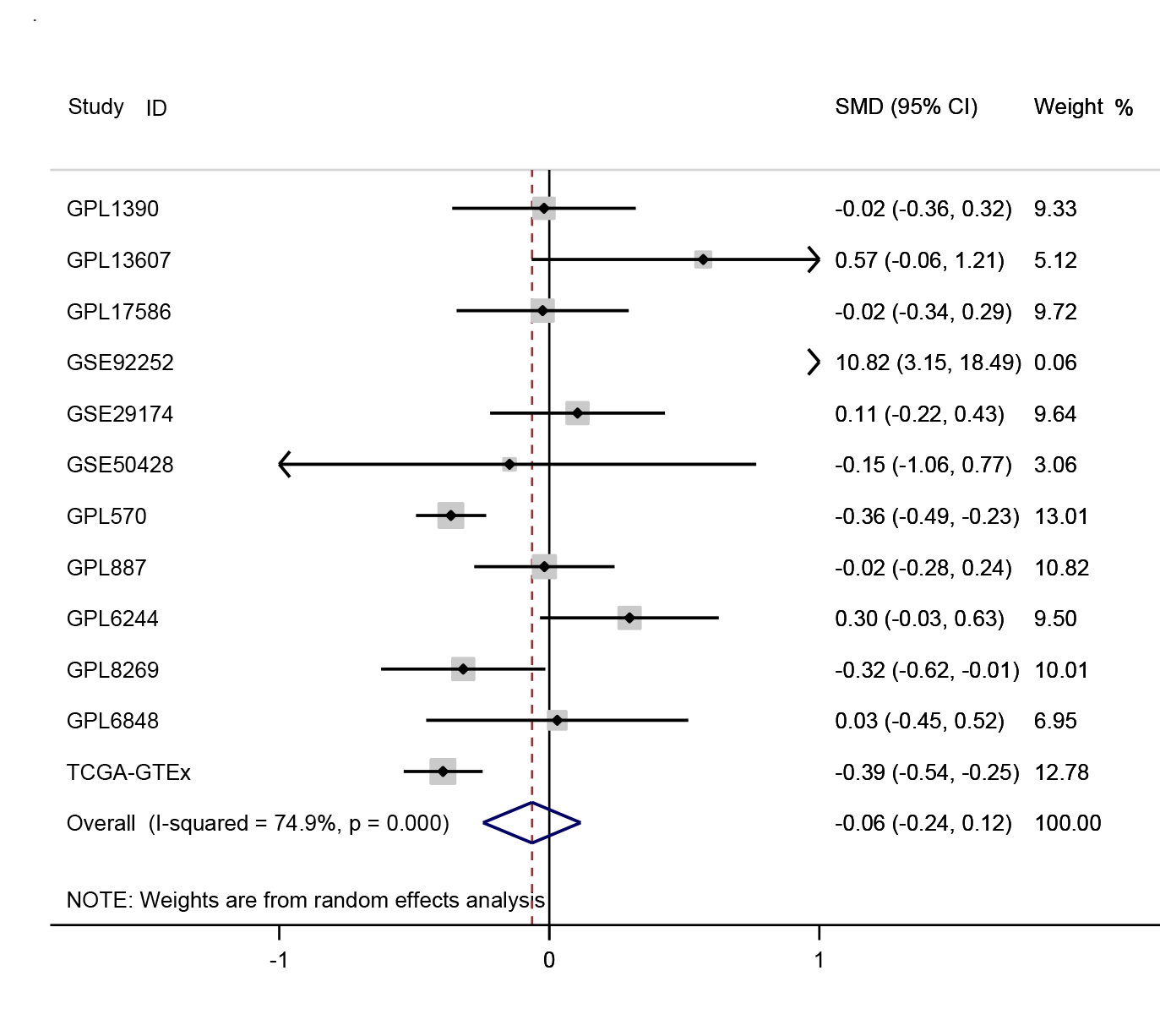

Supplement: Supplementary file 7 — Additional file 7: Figure S7. Calculation of standard mean deviation (SMD) based on COL8A1 expression in Three Negative Breast cancer (TNBC) and non-TNBC tissues. [file 12935_2020_1465_MOESM7_ESM.tif]

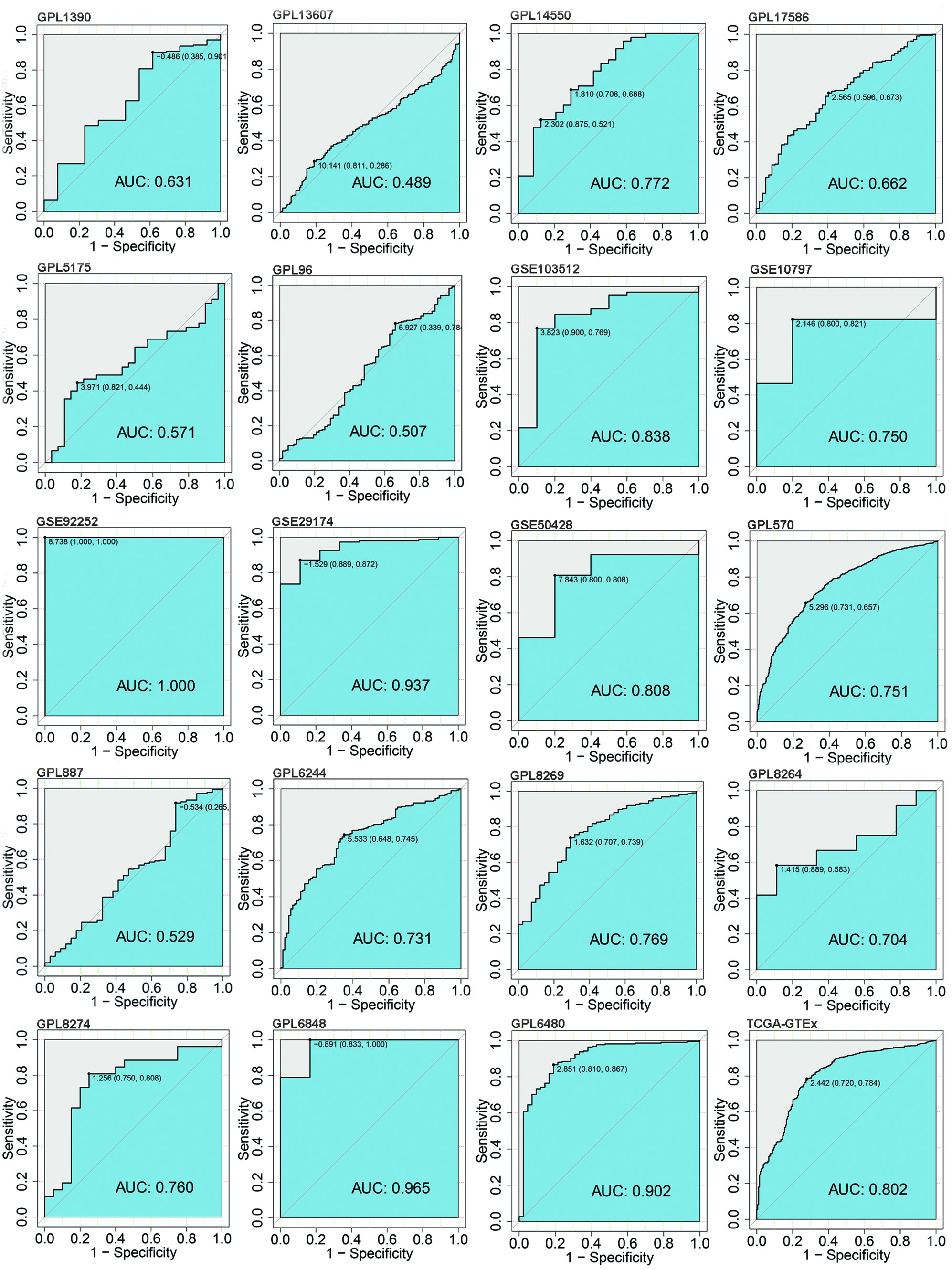

Supplement: Supplementary file 8 — Additional file 8: Figure S8. Receiver operating characteristic (ROC) curves based on COL8A1 expression value in breast cancer (BRCA) patients. An AUC value > 0.70 signified COL8A1 possessed moderate capability in distinguishing BRCA from non-BRCA patients. AUC ,area under the curve [file 12935_2020_1465_MOESM8_ESM.tif]

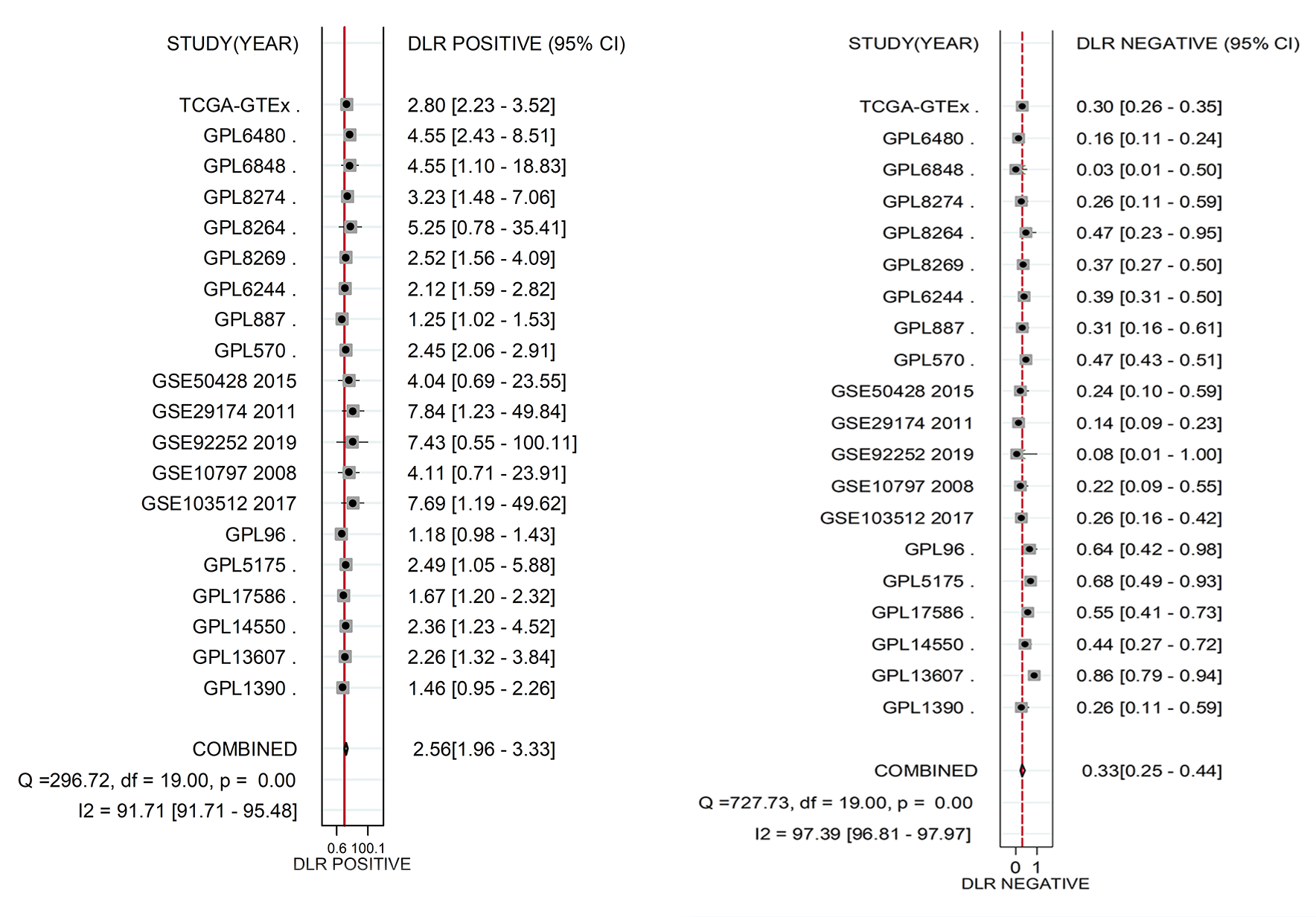

Supplement: Supplementary file 9 — Additional file 9: Figure S9. DLR positive and negative in breast cancer diagnostic trial based on COL8A1 expression level. DLR, Diagnostic likelihood ratio. [file 12935_2020_1465_MOESM9_ESM.tif]

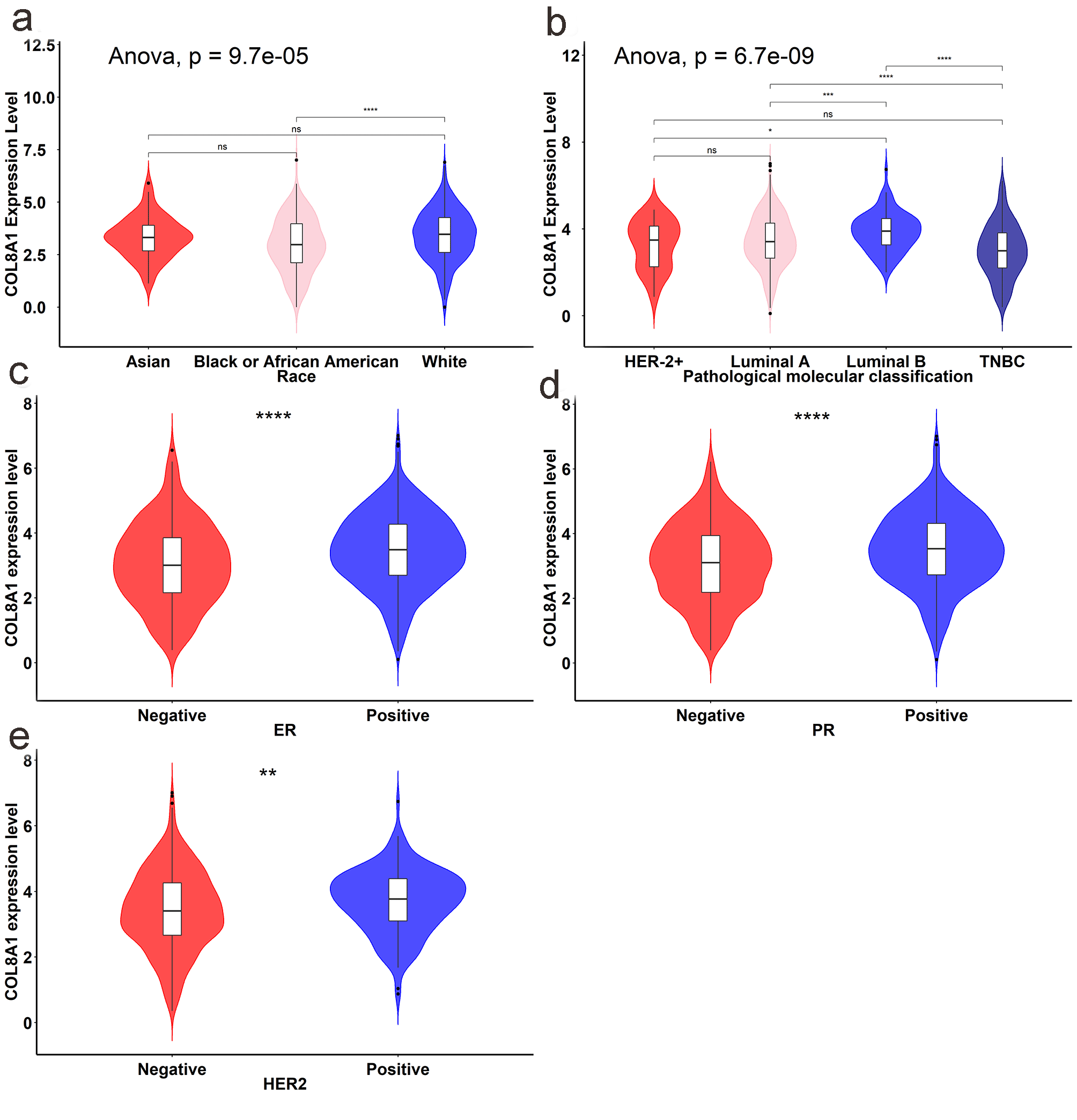

Supplement: Supplementary file 10 — Additional file 10: Figure S10. Association between COL8A1 expression and clinicopathological parameters in breast cancer patients. a Elevated COL8A1 expression correlated with races of breast cancer patients. COL8A1 expression was higher in white compared to Black or African American. b Elevated COL8A1 expression correlated with subtypes of breast cancer. COL8A1 expression was lower in Three Negative Breast Cancer compared to Luminal A or Luminal B subtypes of breast cancer. c Elevated COL8A1 expression correlated with ER status. d Elevated COL8A1 expression correlated with PR status. e Elevated COL8A1 expression correlated with HER-2 status. [file 12935_2020_1465_MOESM10_ESM.tif]

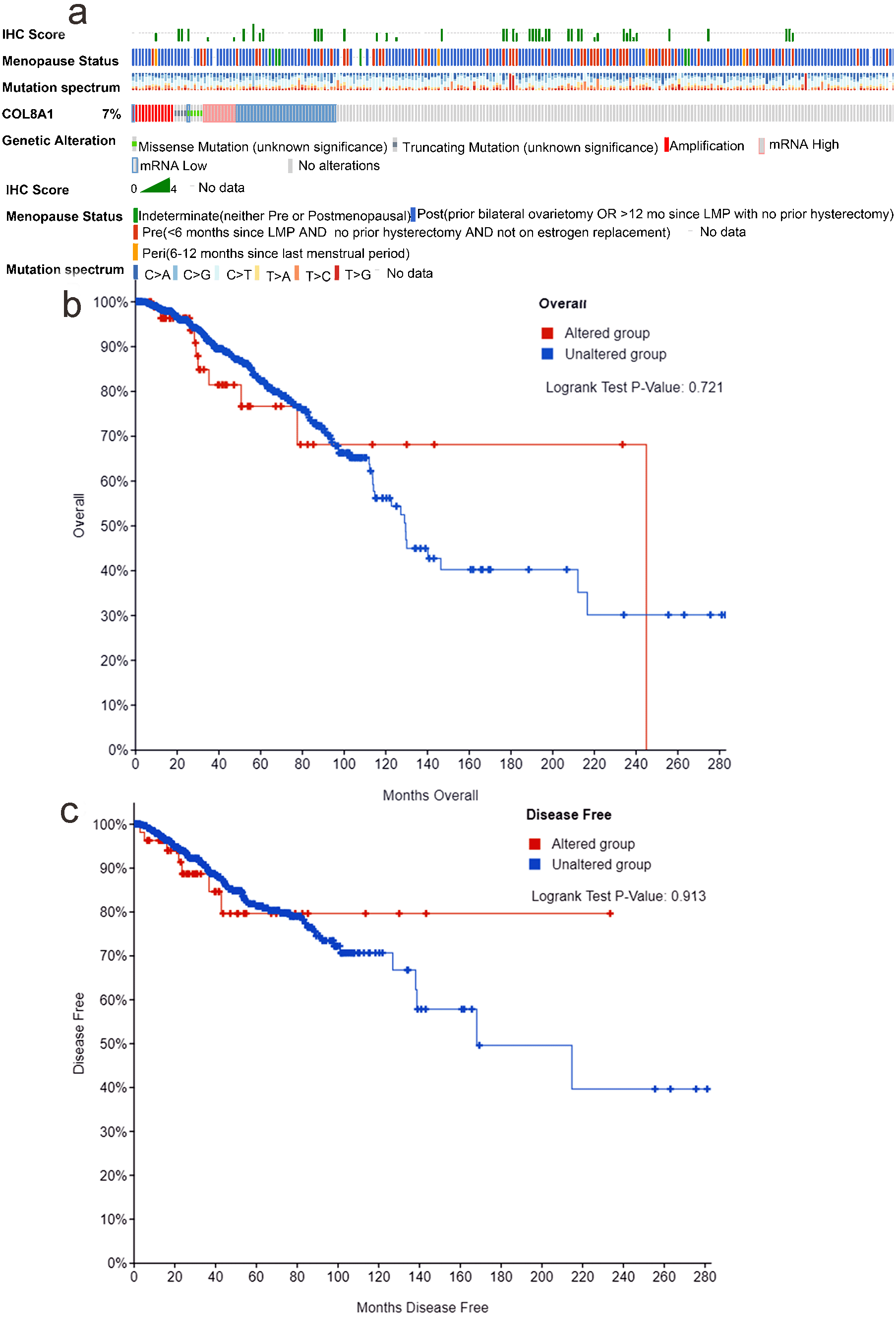

Supplement: Supplementary file 13 — Additional file 13: Figure S11. Genetic alteration types of COL8A1 in breast cancer. a Amplification, mRNA high, and mRNA low were predominant alteration types of COL8A1 in breast cancer. b, c. indicated no significant difference between overall or disease-free survival conditions of COL8A1 altered and unaltered groups in breast cancer. [file 12935_2020_1465_MOESM13_ESM.tif]

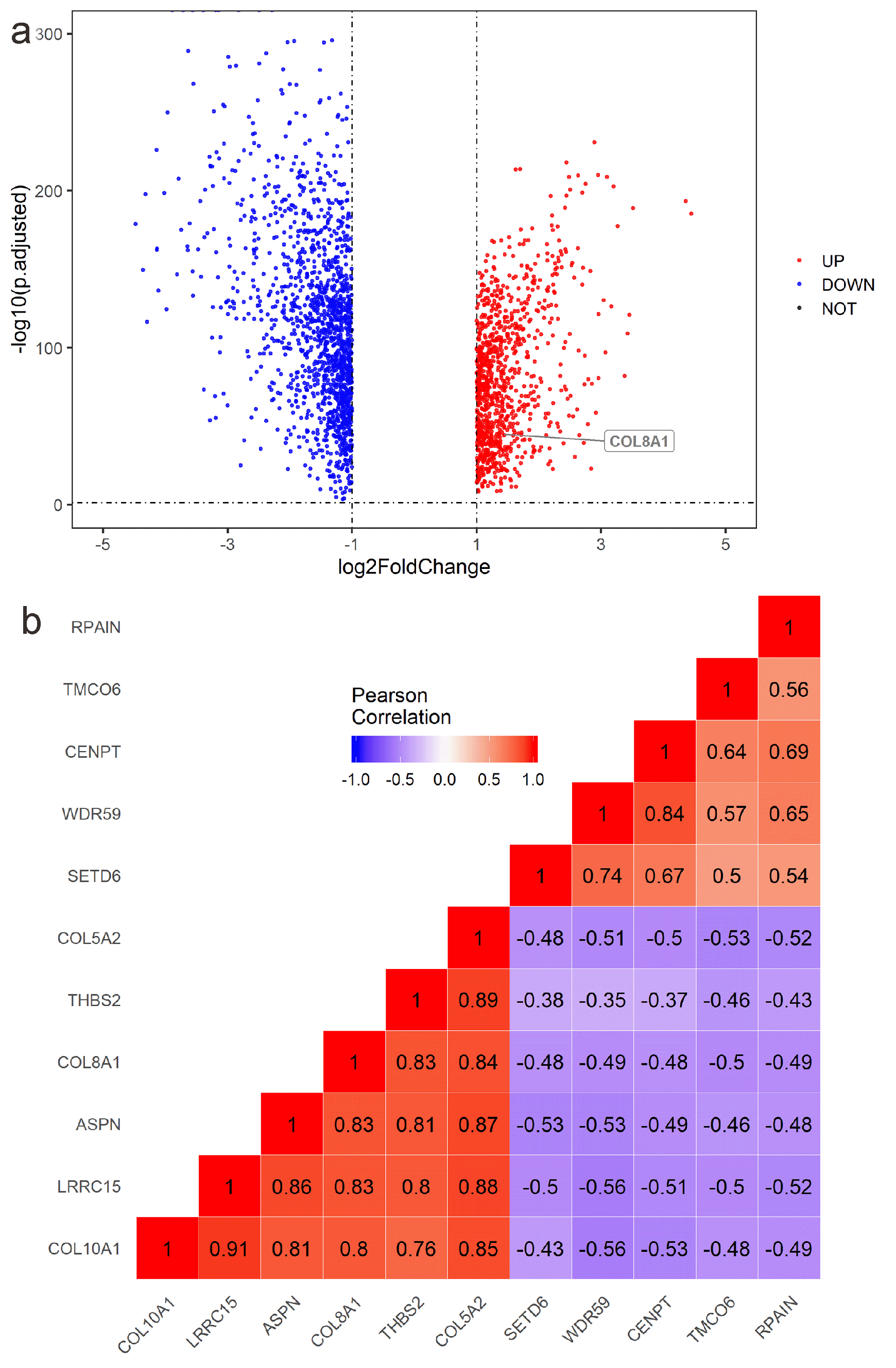

Supplement: Supplementary file 15 — Additional file 15: Figure S12. The identification of DEGs and COL8A1 related CEGs in breast cancer. TCGA dataset was selected to partially show A. DEGs and B. COL8A1 related CEGs. DEGs, differentially expressed genes; CEGs, co-expressed genes. [file 12935_2020_1465_MOESM15_ESM.tif]

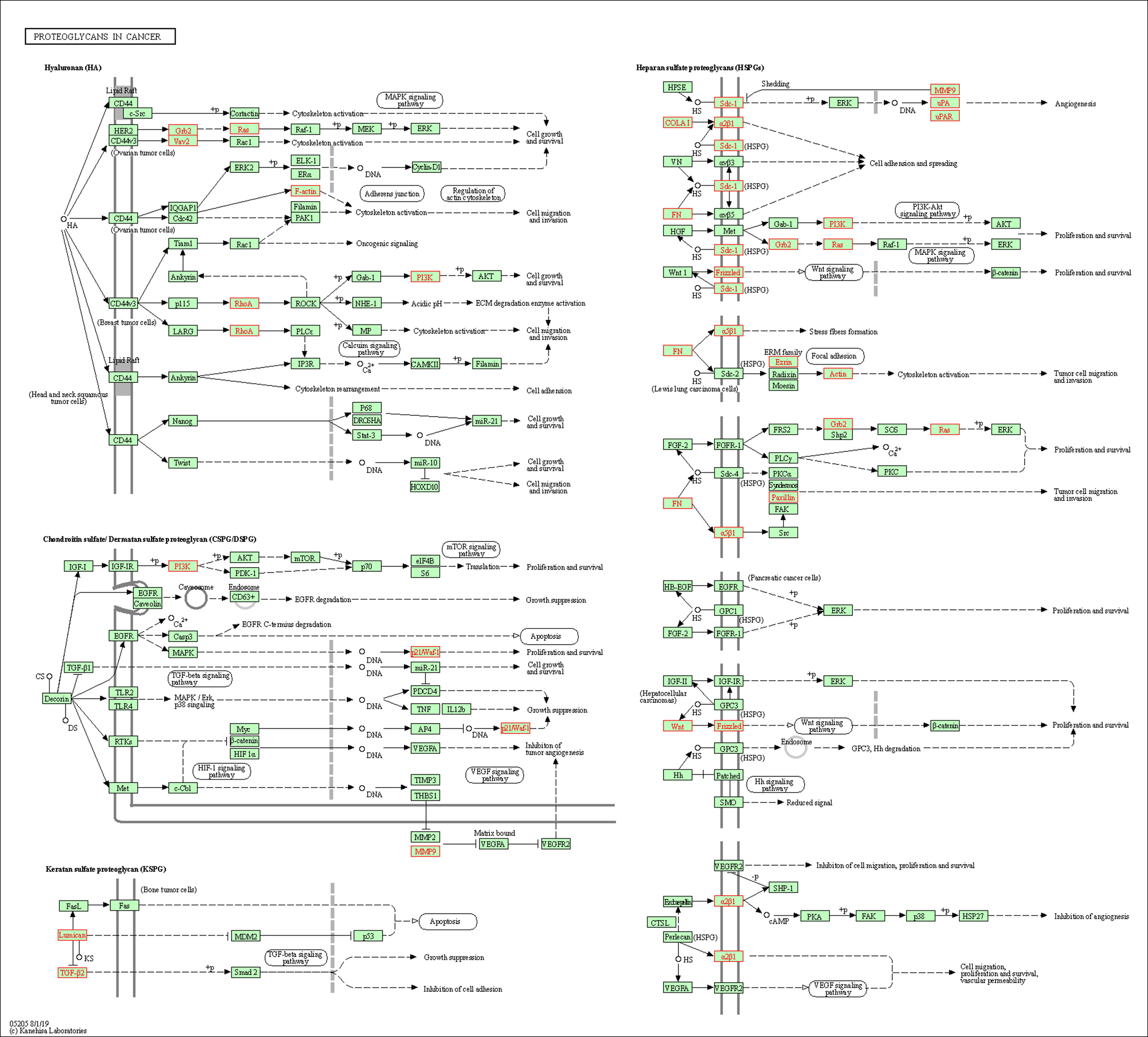

Supplement: Supplementary file 17 — Additional file 17: Figure S13. Intersected DEGs and CEGs positively related to COL8A1 clustered in the proteoglycans in cancer pathways Kyoto Encyclopedia of Genes and Genomes (KEGG) pathway: Proteoglycans in cancer (ID: hsa05205). [file 12935_2020_1465_MOESM17_ESM.tif]

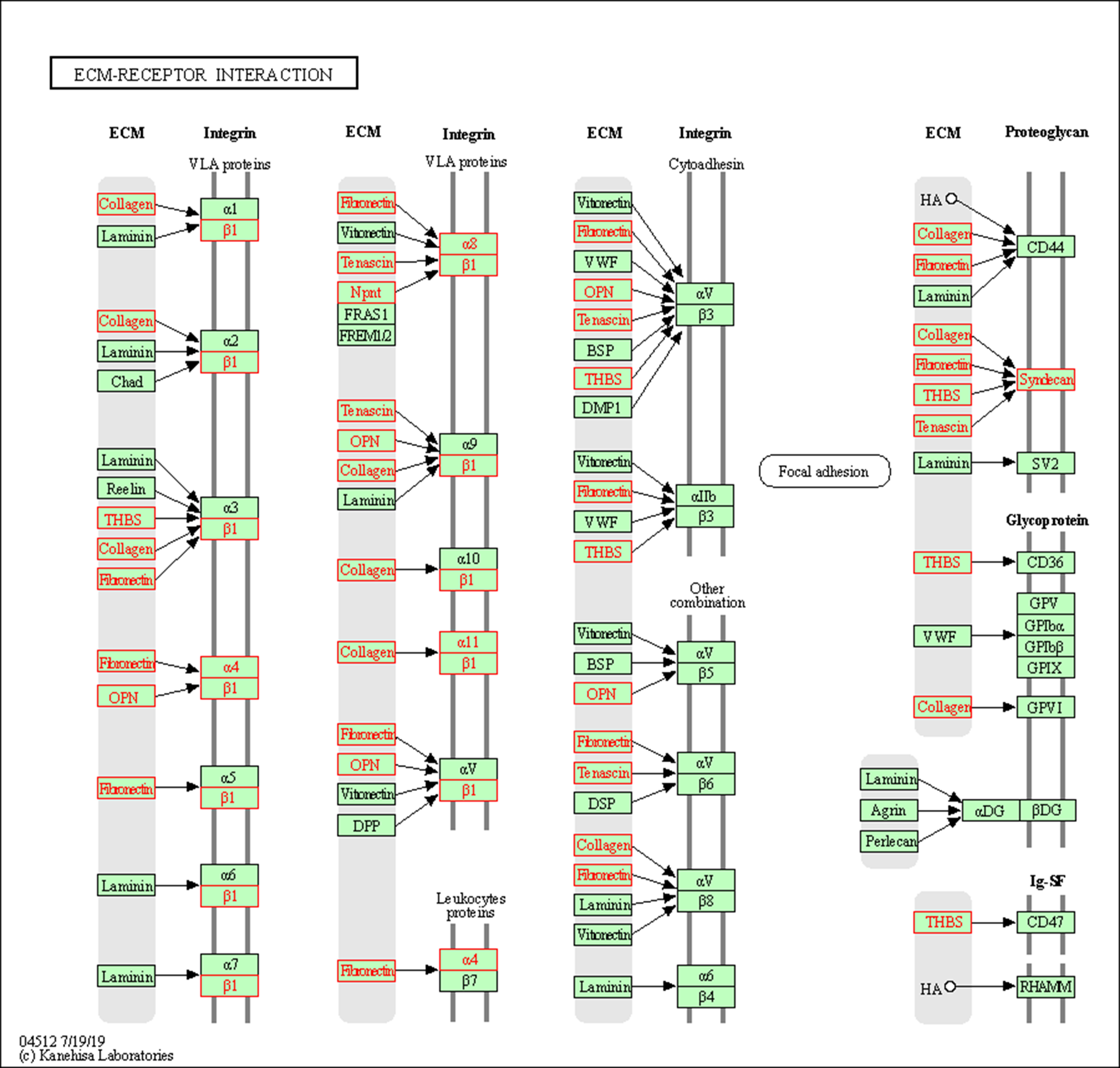

Supplement: Supplementary file 18 — Additional file 18: Figure S14. Intersected DEGs and CEGs positively related to COL8A1 clustered in the ECM-receptor interaction pathways Kyoto Encyclopedia of Genes and Genomes (KEGG) pathway: ECM-receptor interaction (ID: hsa04512). [file 12935_2020_1465_MOESM18_ESM.tif]

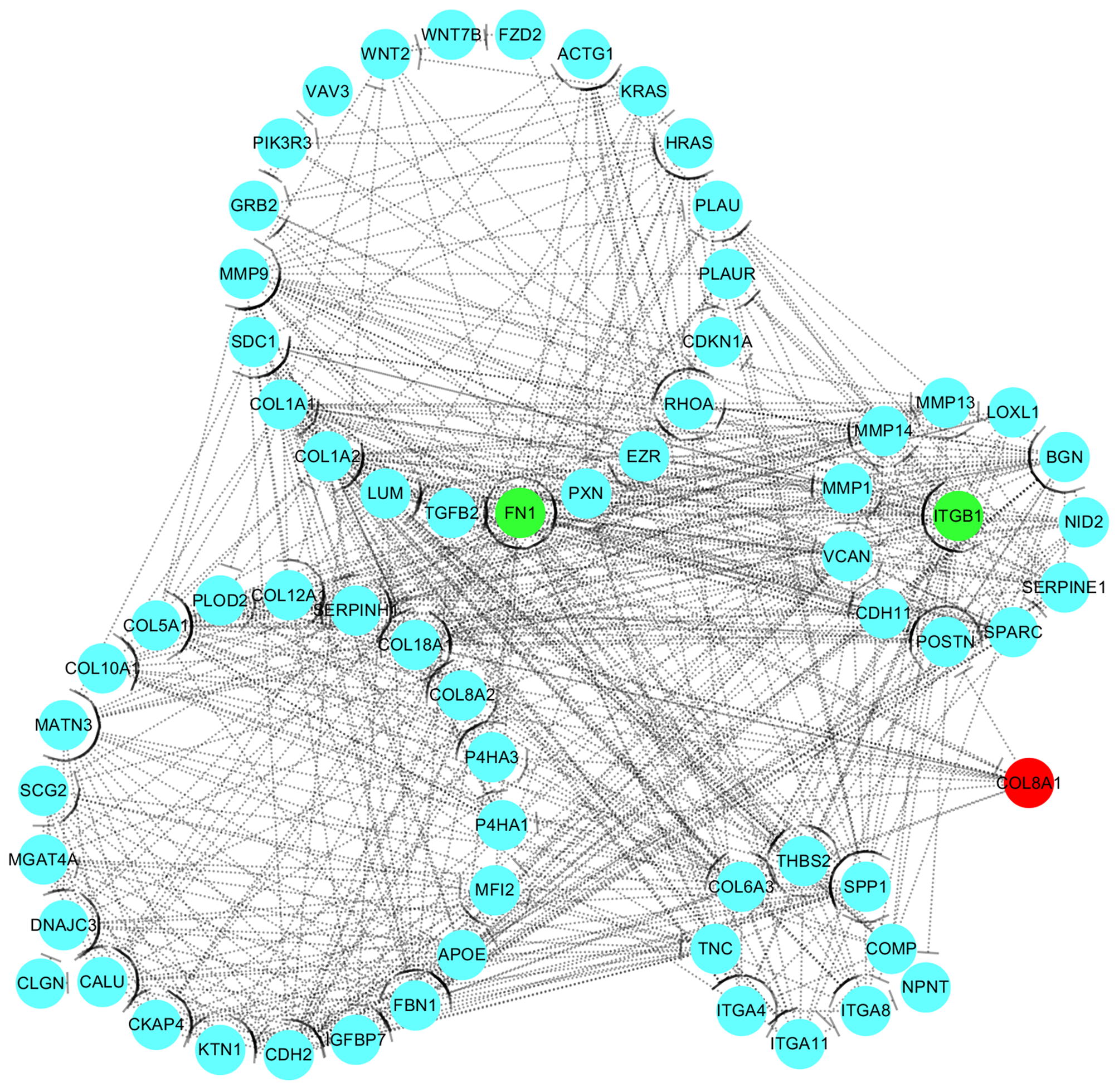

Supplement: Supplementary file 19 — Additional file 19: Figure S15. Co-expressed network of COL8A1 in breast cancer. Genes clustered in the top two KEGG pathways or the top two functional modules were displayed. KEGG, Kyoto Encyclopedia of Genes and Genomes. [file 12935_2020_1465_MOESM19_ESM.tif]
