# Supplementary material for: Clinical value and potential mechanisms of COL8A1 upregulation in breast cancer: a comprehensive analysis
Source: Cancer Cell Int. 2020 Aug 14;20:392. doi: 10.1186/s12935-020-01465-8 (PMC7427770; doi:10.1186/s12935-020-01465-8)
Supplement: Supplementary file 11 — Additional file 11: Table S1. The relevance between COL8A1 expression and clinicopathological parameters of breast cancer patients. Independent-samples t-test or one way analysis of variance (ANOVA) was used to compare the COL8A1 expression level between two groups or more groups, respectively. *A P-value < 0.05 indicates statistically significant. [file 12935_2020_1465_MOESM11_ESM.docx]

Additional file 11: Table S1. The relevance between COL8A1 expression and clinicopathological parameters of breast cancer patients. Independent-samples t-test or one way analysis of variance (ANOVA) was used to compare the COL8A1 expression level between two groups or more groups, respectively. ^*^A *P*-value < 0.05 indicates statistically significant.

| **Clinical features** |  | **Expression level of COL8A1** | | | | |  |
| --- | --- | --- | --- | --- | --- | --- | --- |
|  | **N** | | **M** | **SD** | **t or F** | **df** | ***P*** |
| **Tissue** |  | |  |  |  |  |  |
| BRCA | 1089 | | 3.3557 | 1.18912 | 7.896 | 1200 | 0^*^ |
| Non-cancer | 113 | | 2.4335 | 1.10714 |  |  |  |
| **Tumor status** |  | |  |  |  |  |  |
| With tumor | 93 | | 3.2811 | 1.10351 | -0.366 | 964 | 0.714 |
| Tumor free | 873 | | 3.3285 | 1.19585 |  |  |  |
| **Age (years)** |  | |  |  |  |  |  |
| <50 | 293 | | 3.2888 | 1.14616 | -1.228 | 1075 | 0.22 |
| ≥50 | 784 | | 3.3887 | 1.20389 |  |  |  |
| **Gender** |  | |  |  |  |  |  |
| Male | 12 | | 3.4421 | 0.68774 | 0.433 | 11.751 | 0.673 |
| Female | 1077 | | 3.3547 | 1.19367 |  |  |  |
| **Race** |  | |  |  |  |  |  |
| Asian | 61 | | 3.3175 | 0.97661 | 10.242 |  | 0^*^ |
| Black or African American | 182 | | 2.9929 | 1.27936 |  |  |  |
| White | 751 | | 3.4364 | 1.17951 |  |  |  |
| **Ethnicity** |  | |  |  |  |  |  |
| Hispanic or Latino | 39 | | 3.124 | 1.11329 | -1.148 | 914 | 0.251 |
| Not Hispanic or Latino | 877 | | 3.3502 | 1.20743 |  |  |  |
| **Menopause status** |  | |  |  |  |  |  |
| Premenopause | 226 | | 3.3629 | 1.13393 | 0.317 |  | 0.728 |
| Perimenopause | 39 | | 3.5181 | 1.25705 |  |  |  |
| Postmenopause | 701 | | 3.3648 | 1.19246 |  |  |  |
| **Pathologic stage** |  | |  |  |  |  |  |
| Stage I | 181 | | 3.3985 | 1.17235 | 0.406 |  | 0.804 |
| Stage II | 618 | | 3.3314 | 1.22045 |  |  |  |
| Stage III | 247 | | 3.3806 | 1.10938 |  |  |  |
| Stage IV | 20 | | 3.1719 | 1.24728 |  |  |  |
| **Tumor** |  | |  |  |  |  |  |
| T1 | 279 | | 3.4642 | 1.15875 | 2.011 |  | 0.091 |
| T2 | 630 | | 3.3606 | 1.20372 |  |  |  |
| T3 | 137 | | 3.1321 | 1.13792 |  |  |  |
| T4 | 40 | | 3.3345 | 1.30389 |  |  |  |
| TX | 3 | | 2.7338 | 0.15651 |  |  |  |
| **Lymph node** |  | |  |  |  |  |  |
| N0 | 513 | | 3.3352 | 1.26382 | 1.623 |  | 0.166 |
| N1 | 360 | | 3.348 | 1.12804 |  |  |  |
| N2 | 120 | | 3.5613 | 1.09244 |  |  |  |
| N3 | 76 | | 3.3199 | 1.07425 |  |  |  |
| NX | 20 | | 2.9224 | 1.172 |  |  |  |
| **Metastasis** |  | |  |  |  |  |  |
| M0 | 906 | | 3.3808 | 1.18191 |  |  |  |
| M1 | 22 | | 3.0801 | 1.30519 | 1.401 |  | 0.247 |
| MX | 161 | | 3.2524 | 1.21064 |  |  |  |
| **Pathological molecular classification** | | | |  |  |  |  |
| Luminal A | 570 | | 3.4244 | 1.18156 | 13.946 |  | 0^*^ |
| Luminal B | 97 | | 3.8675 | 0.88022 |  |  |  |
| HER-2+ | 38 | | 3.2648 | 1.11347 |  |  |  |
| TNBC | 227 | | 3.0026 | 1.23157 |  |  |  |
| **ER** |  | |  |  |  |  |  |
| Positive | 808 | | 3.43292 | 1.182417 | 4.490691 | 365.9461 | 9.53E-06^*^ |
| Negative | 239 | | 3.016225 | 1.282294 |  |  |  |
| **PR** |  | |  |  |  |  |  |
| Positive | 699 | | 3.473616 | 1.19364 | 4.992674 | 670.6586 | 7.60E-07^*^ |
| Negative | 344 | | 3.075911 | 1.217219 |  |  |  |
| **HER-2+** |  | |  |  |  |  |  |
| Positive | 164 | | 3.686285 | 0.999905 | 2.850737 | 726 | 0.004486^*^ |
| Negative | 564 | | 3.383637 | 1.247869 |  |  |  |
