# Supplementary material for: Clinical value and potential mechanisms of COL8A1 upregulation in breast cancer: a comprehensive analysis
Source: Cancer Cell Int. 2020 Aug 14;20:392. doi: 10.1186/s12935-020-01465-8 (PMC7427770; doi:10.1186/s12935-020-01465-8)
Supplement: Supplementary file 12 — Additional file 12: Table S2. Clinical data of breast cancer samples used to perform immunohistochemistry. [file 12935_2020_1465_MOESM12_ESM.docx]

Additional file 12: Table S2. Clinical data of breast cancer samples used to perform immunohistochemistry.

| **Clinicopathological features** | **Number of cases** | **Low COL8A1** | **High COL8A1** | **χ^2^** | ***P*** |
| --- | --- | --- | --- | --- | --- |
| **Age(years)** |  |  |  |  |  |
| ≤50 | 73 | 34 | 39 | 0.149 | 0.7 |
| >50 | 42 | 18 | 24 |  |  |
| **Histological grade** |  |  |  |  |  |
| Ⅰ-Ⅱ | 89 | 40 | 49 | 0.012 | 0.913 |
| Ⅲ | 26 | 12 | 14 |  |  |
| **Tumor size(cm)** |  |  |  |  |  |
| ≤2 | 28 | 12 | 16 | 0.544 | 0.762 |
| 2-5 | 69 | 33 | 36 |  |  |
| >5 | 18 | 7 | 11 |  |  |
| **Node** |  |  |  |  |  |
| N0 | 55 | 23 | 32 | 0.492 | 0.483 |
| N1-N3 | 60 | 29 | 31 |  |  |
| **Metastasis** |  |  |  |  |  |
| M0 | 107 | 46 | 61 | 3.079 | 0.079 |
| M1 | 8 | 6 | 2 |  |  |
| **TNM stage** |  |  |  |  |  |
| Ⅰ+Ⅱ | 78 | 37 | 41 | 0.482 | 0.488 |
| Ⅲ+Ⅳ | 37 | 15 | 22 |  |  |
| **Molecular classification** |  |  |  |  |  |
| Luminal A | 12 | 7 | 5 | 4.364 | 0.225 |
| Luminal B | 70 | 35 | 35 |  |  |
| HER-2+ | 18 | 5 | 13 |  |  |
| TNBC | 12 | 4 | 8 |  |  |
| **ER** |  |  |  |  |  |
| Negative | 35 | 10 | 25 | 5.628 | 0.018 |
| Positive | 80 | 42 | 38 |  |  |
| **PR** |  |  |  |  |  |
| Negative | 47 | 18 | 29 | 1.536 | 0.215 |
| Positive | 68 | 34 | 34 |  |  |
| **HER-2+** |  |  |  |  |  |
| Negative | 70 | 36 | 34 | 1.796 | 0.18 |
| Positive | 37 | 14 | 23 |  |  |
| **P53** |  |  |  |  |  |
| Negative | 42 | 16 | 26 | 1.677 | 0.195 |
| Positive | 69 | 35 | 34 |  |  |
| **Ki-67** |  |  |  |  |  |
| <14% | 18 | 9 | 9 | 0.197 | 0.657 |
| **≥**14% | 97 | 43 | 54 |  |  |
