# Supplementary material for: Clinical value and potential mechanisms of COL8A1 upregulation in breast cancer: a comprehensive analysis
Source: Cancer Cell Int. 2020 Aug 14;20:392. doi: 10.1186/s12935-020-01465-8 (PMC7427770; doi:10.1186/s12935-020-01465-8)
Supplement: Supplementary file 14 — Additional file 14: Table S3. Mutation types of COL8A1 in breast cancer patients based on the Catalogue Of Somatic Mutations In Cancer (COSMIC). [file 12935_2020_1465_MOESM14_ESM.docx]

Additional file 14: Table S3. Mutation types of COL8A1 in breast cancer patients based on the Catalogue Of Somatic Mutations In Cancer (COSMIC).

| Position | CDS Mutation | AA Mutation | Legacy Mutation ID | Count | Type |
| --- | --- | --- | --- | --- | --- |
| 161 | c.482G>T | p.G161V | COSM447139 | 1 | Substitution - Missense |
| 207 | c.615_629del | p.I207_G211del | COSM5802385 | 1 | Deletion - In frame |
| 220 | c.659G>C | p.G220A | COSM160151 | 1 | Substitution - Missense |
| 334 | c.1002G>A | p.G334= | COSM447140 | 1 | Substitution - coding silent |
| 521 | c.1562C>T | p.P521L | COSM447141 | 1 | Substitution - Missense |
| 599 | c.1795dup | p.H599Pfs*6 | COSM1417242 | 2 | Insertion - Frameshift |
