# Supplementary material for: Clinical value and potential mechanisms of COL8A1 upregulation in breast cancer: a comprehensive analysis
Source: Cancer Cell Int. 2020 Aug 14;20:392. doi: 10.1186/s12935-020-01465-8 (PMC7427770; doi:10.1186/s12935-020-01465-8)
Supplement: Supplementary file 16 — Additional file 16: Table S4. Functional enrichment based on 632 overlapping genes of upregulated DEGs and CEGs positively related to COL8A1. Only the top four terms or pathways were exhibited. KEGG, Kyoto Encyclopedia of Genes and Genomes; DO, Disease Ontology; GO, Gene Ontology; BP, biological process; CC, cellular component; MF, molecular function; DEGs, differentially expressed genes; CEGs, co-expressed genes. Table S5. Functional enrichment based on 322 overlapping genes of downregulated DEGs and CEGs negatively related to COL8A1. Only the top two terms or pathways were exhibited. KEGG, Kyoto Encyclopedia of Genes and Genomes, GO, Gene Ontology; BP, biological process; CC, cellular component; MF, molecular function. [file 12935_2020_1465_MOESM16_ESM.docx]

Additional file 16: Table S4. Functional enrichment based on 632 overlapping genes of upregulated differentially expressed genes and COL8A1 positively related co-expressed genes. Only the top four terms or pathways were exhibited. KEGG, Kyoto Encyclopedia of Genes and Genomes; DO, Disease Ontology; GO, Gene Ontology; BP, biological process; CC, cellular component; MF, molecular function.

| **ID** | **Description** | **Gene Ratio** | **Bg Ratio** | **p-value** | **p.adjust** | **q-value** | **Count** |
| --- | --- | --- | --- | --- | --- | --- | --- |
| **KEGG** |  |  |  |  |  |  |  |
| hsa04145 | Phagosome | 21/328 | 152/8018 | 8.49E-07 | 0.000253 | 0.000198 | 21 |
| hsa05205 | Proteoglycans in cancer | 23/328 | 204/8018 | 8.99E-06 | 0.000651 | 0.000509 | 23 |
| hsa05220 | Chronic myeloid leukemia | 13/328 | 76/8018 | 1.05E-05 | 0.000651 | 0.000509 | 13 |
| hsa04512 | ECM-receptor interaction | 14/328 | 88/8018 | 1.15E-05 | 0.000651 | 0.000509 | 14 |
| **DO** |  |  |  |  |  |  |  |
| DOID:9538 | multiple myeloma | 36/376 | 264/8007 | 5.59E-09 | 5.65E-06 | 4.16E-06 | 36 |
| DOID:0070004 myeloma | | 40/376 | 323/8007 | 1.28E-08 | 6.47E-06 | 4.76E-06 | 40 |
| DOID:4960 | bone marrow cancer | 40/376 | 328/8007 | 1.98E-08 | 6.69E-06 | 4.92E-06 | 40 |
| DOID:854 | collagen disease | 29/376 | 201/8007 | 5.23E-08 | 1.32E-05 | 9.73E-06 | 29 |
| **Reactcome** | |  |  |  |  |  |  |
| R-HSA-1474244 | Extracellular matrix organization | 55/428 | 301/10619 | 8.02E-22 | 8.24E-19 | 7.52E-19 | 55 |
| R-HSA-3000178 | ECM proteoglycans | 21/428 | 76/10619 | 1.19E-12 | 6.12E-10 | 5.58E-10 | 21 |
| R-HSA-216083 | Integrin cell surface interactions | 20/428 | 85/10619 | 1.03E-10 | 3.53E-08 | 3.22E-08 | 20 |
| R-HSA-1474228 | Degradation of the extracellular matrix | 25/428 | 140/10619 | 2.78E-10 | 7.15E-08 | 6.52E-08 | 25 |
| **GO BP** |  |  |  |  |  |  |  |
| GO:0030198 | extracellular matrix organization | 60/573 | 334/17913 | 3.72E-28 | 1.69E-24 | 1.41E-24 | 60 |
| GO:0043062 | extracellular structure organization | 64/573 | 387/17913 | 7.35E-28 | 1.69E-24 | 1.41E-24 | 64 |
| GO:0030199 | collagen fibril organization | 16/573 | 45/17913 | 2.68E-13 | 4.11E-10 | 3.41E-10 | 16 |
| GO:0001503 | ossification | 40/573 | 388/17913 | 7.28E-11 | 8.38E-08 | 6.96E-08 | 40 |
| **GO CC** |  |  |  |  |  |  |  |
| GO:0031012 | extracellular matrix | 60/591 | 468/18678 | 1.58E-20 | 5.88E-18 | 4.74E-18 | 60 |
| GO:0062023 | collagen-containing extracellular matrix | 55/591 | 399/18678 | 2.30E-20 | 5.88E-18 | 4.74E-18 | 55 |
| GO:0005788 | endoplasmic reticulum lumen | 35/591 | 306/18678 | 5.13E-11 | 8.75E-09 | 7.05E-09 | 35 |
| GO:0030055 | cell-substrate junction | 38/591 | 404/18678 | 2.27E-09 | 2.90E-07 | 2.34E-07 | 38 |
| **GO MF** |  |  |  |  |  |  |  |
| GO:0005201 | extracellular matrix structural constituent | 34/566 | 158/16969 | 2.04E-18 | 1.42E-15 | 1.26E-15 | 34 |
| GO:0005518 | collagen binding | 15/566 | 61/16969 | 1.00E-09 | 3.48E-07 | 3.07E-07 | 15 |
| GO:0005178 | integrin binding | 18/566 | 113/16969 | 3.54E-08 | 8.22E-06 | 7.26E-06 | 18 |
| GO:0019864 | IgG binding | 6/566 | 10/16969 | 2.51E-07 | 4.37E-05 | 3.86E-05 | 6 |

Additional file 15: Table S5. Functional enrichment based on 322 overlapping genes of downregulated differentially expressed genes and COL8A1 negatively related co-expressed genes. Only the top two terms or pathways were exhibited. KEGG, Kyoto Encyclopedia of Genes and Genomes; GO, Gene Ontology; BP, biological process; CC, cellular component; MF, molecular function.

| **ID** | **Description** | **Gene Ratio** | ***P*-value** | ***P*.adjust** | **q value** | **Count** |
| --- | --- | --- | --- | --- | --- | --- |
| **KEGG** |  |  |  |  |  |  |
| hsa00310 | Lysine degradation | 2/6 | 0.025849 | 0.20679 | 0.190464 | 2 |
| hsa00512 | Mucin type O-glycan biosynthesis | 1/6 | 0.131896 | 0.31917 | 0.293972 | 1 |
| **GO BP** |  |  |  |  |  |  |
| GO:2000052 | positive regulation of non-canonical Wnt signaling pathway | 2/57 | 0.000643 | 0.509185 | 0.496968 | 2 |
| GO:0060192 | negative regulation of lipase activity | 2/57 | 0.00116 | 0.509185 | 0.496968 | 2 |
| **GO CC** |  |  |  |  |  |  |
| GO:0005840 | ribosome | 4/68 | 0.013427 | 0.707586 | 0.696774 | 4 |
| GO:0031225 | anchored component of membrane | 3/68 | 0.019283 | 0.707586 | 0.696774 | 3 |
| **GO MF** |  |  |  |  |  |  |
| GO:0005544 | calcium-dependent phospholipid binding | 2/58 | 0.00354 | 0.335079 | 0.330105 | 2 |
| GO:0016879 | ligase activity, forming carbon-nitrogen bonds | 2/58 | 0.00824 | 0.335079 | 0.330105 | 2 |
